# Potato: from functional genomics to genetic improvement

**DOI:** 10.1186/s43897-024-00105-3

**Published:** 2024-08-19

**Authors:** Li Qu, Xueqing Huang, Xin Su, Guoqing Zhu, Lingli Zheng, Jing Lin, Jiawen Wang, Hongwei Xue

**Affiliations:** 1 Shanghai Collaborative Innovation Center of Agri-Seeds, Joint Center for Single Cell Biology, School of Agriculture and Biology, Shanghai Jiao Tong University, Shanghai, 200240 China; 2 Guangdong Laboratory for Lingnan Modern Agriculture, College of Agriculture, South China Agricultural University, Guangzhou, 510642 China

**Keywords:** Potato, Vegetative growth, Flowering and senescence, Storage compound, Biotic and abiotic stress, Trait improvement

## Abstract

Potato is the most widely grown non-grain crop and ranks as the third most significant global food crop following rice and wheat. Despite its long history of cultivation over vast areas, slow breeding progress and environmental stress have led to a scarcity of high-yielding potato varieties. Enhancing the quality and yield of potato tubers remains the ultimate objective of potato breeding. However, conventional breeding has faced challenges due to tetrasomic inheritance, high genomic heterozygosity, and inbreeding depression. Recent advancements in molecular biology and functional genomic studies of potato have provided valuable insights into the regulatory network of physiological processes and facilitated trait improvement. In this review, we present a summary of identified factors and genes governing potato growth and development, along with progress in potato genomics and the adoption of new breeding technologies for improvement. Additionally, we explore the opportunities and challenges in potato improvement, offering insights into future avenues for potato research.

## Introduction

Potato (*Solanum tuberosum L.*), a member of the *Solanacea *family, is believed to have originated in the Andes Mountains along the Peru-Bolivia border in South America approximately 10,000 years ago (Spooner et al. 2005). As the world’s third largest food crop after rice and wheat, the cultivation of potatoes has expanded in recent years. By 2020, potatoes were cultivated across 140 countries on a land expanse of 16.5 million hectares, yielding a production of 359 million tons. Potato tubers are rich in starch, high-quality protein, dietary fiber, vitamins (B1, B6, B9, C, and E), minerals (sodium, magnesium, potassium, zinc, iron, and copper), and various bioactive compounds (carotenoids, anthocyanins, phenolics, and flavonoids), offering substantial nutritional benefits (Camire et al. 2009). Potato is a staple food for approximately two-thirds of the global population, plays a crucial role in addressing regional food shortages, combating poverty, and enhancing food security. In addition to their dietary importance, potatoes have various industrial applications, being processed into animal feed, alcohol, and biofuels. Furthermore, potato starch is widely used as a thickener and stabilizer in the food industry as well as a raw material in the production of paper, cosmetics, adhesives, textiles, and plastics (Burlingame et al. 2009; Dupuis and Liu 2019).

As vital crops for food and as vegetables, tubers serve as the primary nutrient storage and reproductive organs, with their yield and quality predominantly determined by their development and maturation processes. The induction and formation of potato tubers are affected by interactions among various environmental factors, plant hormones, and signaling molecules, along with the regulation of numerous essential genes and several signal transduction and metabolic pathways. In-depth research into these key factors and the elucidation of tuberization mechanisms are crucial for advancing the yield and quality of potato tubers (Agrawal et al. 2008).

Although extensive climate adaptation has promoted the widespread distribution of potatoes globally, potatoes are vulnerable to various abiotic stresses (nutrient deficiency, cold/frost, heat, drought, salinity, and flooding) and biotic stresses (fungal, bacterial, viral, and insect pests), along with post-harvest problems (such as enzymatic browning caused by injury and the accumulation of reducing sugars during cold storage) (Handayani et al. 2019), all of which can compromise the quality and yield of potato tubers. Thus, there is a pressing need to develop elite potato varieties with enhanced agronomic traits and resistance to biotic and abiotic stresses through accelerated breeding methods, which is crucial for economic and agricultural sustainability.

Most potatoes grown commercially are tetraploid cultivated varieties. Due to their complex tetraploid inheritance, highly heterozygous genome, self-incompatibility, and inbreeding depression, genetic breeding and variety improvement in potatoes have been challenging. However, advances in molecular biology, including rapid progress in sequencing technology, genomic selection, and multi-omics approaches, have spurred the development of comparative genomic analysis and functional research on potato genes. Furthermore, availability of the potato genome sequence and efficient potato transformation systems have substantially advanced potato genetic engineering, enhancing key agronomic traits. This review highlights recent progress in potato functional genomics and genetic engineering, along with their applications in potato breeding over the past decade. In addition, we discuss the challenges and prospects for future research in potato functional genomics.

### Key regulatory factors involved in potato growth and development and crucial agronomic traits

#### Vegetative growth

Vegetative growth of potato refers to the process of establishment and growth of vegetative organs, such as roots, stems, and leaves. We categorized potato growth and development into six key phases: (a) dormancy stage, where freshly harvested tubers experience a period of dormancy with inhibited visible bud growth; (b) tuber sprouting stage, where tubers transition from dormancy to sprouting, with sprouts developing from the eyes of the tuber; (c) vegetative growth stage, starting with sprout formation and continuing until 8–12 leaves are formed, alongside root system and stolon development; (d) tuber induction and initiation, beginning with tuber emergence at the stolon ends and extending until the leaf system is fully developed; (e) tuber development, marked by substantial tuber elongation and growth cessation in the vegetative and root systems; and (f) tuber maturation, involving leaf structure physiological aging and the start of tuber skin tightening and thickening (Saidi and Hajibarat  2021).

#### Dormancy of tubers

The dormancy and germination of potato tubers are crucial for potato cultivation, production, and processing because tubers without a dormancy period are difficult to sprout (Haider et al. 2019; Haider et al. 2022). Tuber dormancy prevents seed potatoes harvested in spring or summer from sprouting too soon planted in the following summer and autumn (Saidi and Hajibarat 2021). Moreover, when tubers are used as food and processing materials, a prolonged dormancy period is necessary for transportation and storage. Premature dormancy break can lead to substantial water and nutrient consumption, reducing commodity quality and value. Controlling the dormancy phase duration poses a major challenge in the potato industry and for seed producers (Mouzo et al. 2022).

Understanding molecular mechanisms underlying potato tuber development is essential for enhancing yield and quality. Recent advancements in molecular biology have highlighted the complex role of phytohormones in potato tuber development, including their involvement in dormancy and sprouting regulation. Recently, a comparative proteome profiling of potato cultivars during endodormancy was performed through a high-resolution two-dimensional electrophoresis (2-DE) coupling with reversed-phase liquid chromatography-tandem mass spectrometry (LC-Triple TOF MS/MS), identifying a mitochondrial ADP/ATP carrier, catalase isozyme 2, and heat shock 70 kDa protein as key responders to changes in dormancy (Mouzo et al. 2022), and providing valuable insights into the molecular basis of dormancy regulation in potatoes and implications for storage.

Phytohormones are pivotal for regulating potato tuber dormancy and sprouting. Gibberellic acid (GA), cytokinins (CKs), and auxin contribute to dormancy termination (Muthoni et al. 2014), whereas ethylene (ET) and abscisic acid (ABA) help maintain bud dormancy in potatoes (Wróbel et al. 2017). The molecular mechanisms underlying these hormonal interactions are complex and part of an intricate network operating at multiple levels, including signal perception, transduction, transcriptional regulation, and metabolic exchange. Recent studies have indicated the importance of *Auxin response factor* (*ARF*) genes in meristem reactivation and tuber sprouting and the inhibitory effect of strigolactones on tuber bud growth (Liao et al. 2018; Suttle 2004). Additionally, the complete life cycle of potato depends on the orchestrated interaction among various phytohormones, elucidating these molecular interactions is essential crucial for enhancing yield and quality (Aksenova et al. 2012; Saidi and Hajibarat 2021).

Auxin plays a critical role in affecting tuber dormancy and sprout growth. *StARF1 *is downregulated in dormant tubers and upregulated in sprouting ones, and involves in tuber and sprouting dormancy breakage (Ben Chaabane et al. 2013; Liu et al. 2012). The content of free indole-3-acetic acid (IAA) in potato tuber buds increases markedly during germination, associating with the transcription level of *ARF6 *(Gao et al. 2016; Kumlay 2014). In addition, the interplay between auxin and ABA signaling is essential for the shift from dormancy to seed growth (Aksenova et al. 2013). *ARF10* and *ARF16* are necessary for sustaining *ABI3* expression by acting as activators of *ABI3 *transcription (Liu et al. 2012). Transcription factors ABI3, ABI4, and ABI5 are pivotal in the dormancy-to-sprouting transition, controlling the expression of downstream target genes involved in seed germination (Shu et al. 2016). StABI5 plays a crucial role in the regulation of tuber dormancy by controlling auxin signaling pathway and regulating the expression of downstream genes (Zhu et al. 2020a, b). Other gene families involving in ABA metabolism and biosynthesis pathways such as *StZEP*, *StNCED*, *StCYP*, and *StSUT4*, also play important roles during potato dormancy (Gong et al. 2021; Destefano-Beltrán et al. 2006). Transgenic potato plants expressing *CK oxidase/dehydrogenase 1* (*CKX1*) and *IPT*genes showed altered endogenous CK levels and GA-mediated sprouting (Sonnewald and Sonnewald 2014).

Brassinosteroids (BRs) also play crucial roles in regulating plant growth and germination, and it is reported that BR at 500 nM is able to break the dormancy of tubers. Overexpression of *StBIN2*, the negative regulator of BR signal transduction, prolongs dormancy, whereas the silencing *StBIN2 *induces premature sprouting (Liu et al. 2024). RNA-sequencing (RNA-seq) analysis indicated that *StBIN2* affects ABA signal transduction and the expression of lignin synthesis genes, highlighting interactions among *StBIN2*, *StSnRK2.2*, and *StCCJ9 *(Liu et al. 2024).

#### Germination of tubers

Breeding potato cultivars with extended dormancy and decreased sprout growth is crucial for enhancing storage and shelf-life. However, rapid emergence is vital for the seed potato industry. Understanding the molecular mechanisms will facilitate the development of improved cultivars (Sharma et al. 2021). Various factors including plant hormones, genetic makeup, signaling molecules, genotype, storage temperature, and other environmental conditions affect the tuber sprouting (Wang et al. 2020). Using transcriptomic and metabolomic methods, a recent study revealed that chlorine dioxide (ClO_2_) treatment regulated the expression of 3,119 genes and 932 metabolites in potato tubers. Genes downregulated were mainly related to hormone signaling, whereas upregulated ones were primarily involved in processes such as phenylpropanoid biosynthesis and the MAPK signaling pathway (Zheng et al. 2022), providing new insights into the ClO_2_-mediated suppression of potato sprouting. In addition, *StCYP707A* was found to be upregulated in tubers with low T6P levels, accelerating dormancy release (Debast et al. 2011). *GPT2* plays a key role in potato tuber sweetening by transporting G6P across the plasma membrane for dephosphorylation (Barrera-Gavira 2021).

Regulating bud growth through sucrose synthesis and transport is essential for germination. Targeting alkaline invertases (AI) and neutral invertases (NI) genes affects the accumulation of reducing sugars hence to affect germination. The NI genes *StNI4*, *StNI5*, and *StNI6 *promote sucrose breakdown in cold-stored potato tubers, leading to increased accumulation of reducing sugars (Datir and Regan 2022). Exogenous ET is involved in potato sprout regulation, and ET suppresses tuber sprouting, stimulates sucrose synthesis, and inhibits starch regeneration (Dai et al. 2016). Moreover, ET treatment reduces the expression of the invertase inhibitor factor while enhancing the activity of SPS, BAM, AI, and NI (Tosetti et al. 2021), or reduces total phenol accumulation and enhances their degradation, thus inhibiting sprouting (Dako et al. 2021).

**Fig. 1:**
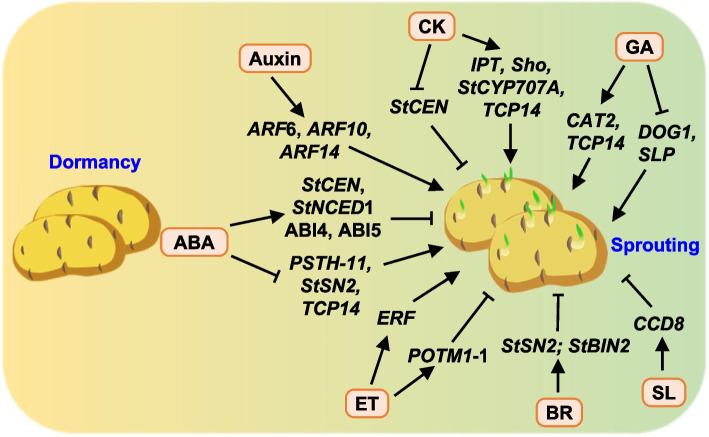
Schematic representation of phytohormone-related pathways in the regulation of potato dormancy and sprouting. Hormones abscisic acid (ABA), cytokinin (CK), ethylene (ET), gibberellins (GAs), auxin, brassinosteroids (BR), and strigolactones (SL) regulate potato dormancy and sprouting synergistically or antagonistically by regulating the expression of related genes

Strigolactones (SLs) and auxin collaboratively inhibit tuber bud growth, affecting shoot and root architecture (Liao et al. 2018). Transgenic potatoes with decreased expression of SL biosynthetic gene *CCD8 *sprouted earlier than wild-type tubers (Pasare et al. 2013). Overexpression of *GA2-oxidase 1* (*StGA2ox1*) reduced early sprout growth, leading to a dwarfed plant phenotype, suggesting that *StGA2ox1 *is important in sprouting initiation instead of dormancy regulation (Kloosterman et al. 2007; Roumeliotis et al. 2013a, b). These findings expanded the understanding of the molecular mechanisms involved in potato tuber dormancy and sprouting (Fig. 1), shedding light on the potential in improving potato storage, quality, and industry efficiency.

#### Overall growth of potato tissues

Potato development from a seed or tuber is a complex process involving asexual reproduction through tuber propagation and sexual reproduction through flower pollination. The root provides nutrients and water for stolon and tuber growth (Iwama 2008). Drought substantially affects yield, especially in shallow-rooted crops such as potatoes (Sołtys-Kalina et al. 2016). The potato root system, known for its shallow depth and weak soil penetration, is considered less efficient than that of other crops (Joshi et al. 2016; Stalhman et al. 2007).

Commercial potatoes are propagated using freshly sprouting tubers, termed “mother” tubers. “Basal roots” first emerge where the sprout meets the mother tuber (Wishart et al. 2013). As the sprout grows, roots develop at specific nodes on its underground stem, closely associated with stolon initiation sites, forming a pattern where four roots encircle the emerging stolon (Joshi et al. 2016). Roots at the stolon/stem junction and nodes on the stolon are termed “stolon roots” and “stolon node roots”, respectively (Wishart et al. 2013). Root system architecture (RSA) is affected by the growth and branching of adventitious roots (ARs) and lateral roots (LRs) (Joshi et al. 2016).

Endogenous and environmental factors regulate the complex adventitious rooting process in potatoes, affecting root elongation, branching, and longevity (Joshi and Ginzberg 2021). Potato plants form adventitious roots post-embryonically, with lateral roots emerging through auxin-dependent cell cycle activation (Joshi et al. 2016). Genes found in model plants and involved in root system responses to environmental cues, such as *ANR1*, *NRT1*, *PHO1*, and *RSA1*, are expressed in potato roots as well, indicating the potential to enhance soil utilization and yield through manipulating potato RSA (Joshi et al. 2016). miR164 affects LR development by targeting NAC transcription factors in potatoes (Zhang et al. 2018). Two *ARF* genes, *StARF10* and *StARF16*, identified as the targets of stu-miR160a/b, exhibited varying expression levels correlated with root development stages (Yang et al. 2021a, b). Suppressing stu-miR8006-p5-1ss9AT considerably altered potato root architecture through affecting its target, auxin-induced in root cultures protein 12 (Duan et al. 2023).

**Fig. 2:**
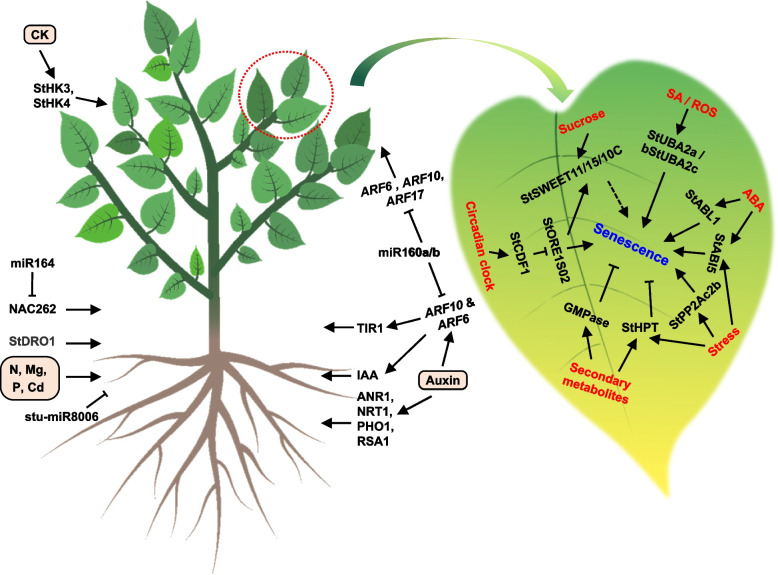
Regulation of potato vegetative growth by phytohormones and inorganic elements, and leaf senescence by environmental stimuli. Genes *ANR1*, *NRT1*, and *PHO1*, and inorganic nutrient elements nitrogen, magnesium, and phosphorus, regulate root development of potato. MicroRNAs and phytohormones regulate leaf development. Circadian clock, SA/ROS, ABA, stress, secondary metabolites, and sucrose regulate leaf senescence through various pathways. StABL1 and StABI5, ABA signalling pathway transcription factors; StUBA2a/b and StUBA2c, the NA-binding proteins; StSWEET11/15/10C, sucrose transporters

Nitrogen (N) is crucial for potato growth, affecting metabolism, photosynthesis, starch synthesis, and antioxidant content, with cultivars responding differently to N availability, highlighting the importance of N management (Zhang et al. 2020). Magnesium (Mg) transport and distribution are vital for root growth, with Mg transporter gene expression varying in response to Mg levels, influencing root development in Mg-deficient conditions (Koch et al. 2020). Phosphorus (P) availability affects the RSA of potato and the expression of genes related to root development. The *StCAD* gene family plays a key role in potato’s response to Cd stress, with post-Cd stress analysis revealing upregulation in most family genes, enhancing CAD activity and promoting lignin accumulation in roots (Fig. 2, left; Yang et al. 2024).

Potato is sensitive to water and nutrient stresses due to its shallow and sparse root system, and lower soil nutrient or water availability limit the growth and tuber yield of potato  (Nurmanov et al. 2019; Milroy et al. 2019). Farmers usually increase fertilizer input to ensure the soil nutrient supply and potato productivity (Xu et al. 2019; Li et al. 2020), which generates gaseous emission and causes environmental pollution, as well as human health hazards (Wang et al. 2020). Enhancing nutrient use efficiency is thus a critical need for achieving sustainable agriculture and human health. With the efforts of scientific researchers, advancements in nutrient utilization and fertilizer management optimization appeared, including Nutrient expert system (Sha et al. 2022), sustainable agri-food systems using multi-nutrient fertilizers in Kenyan smallholder farming systems (Adolwa et al. 2023), and nitrogen management indicators under drip irrigation (Di et al. 2024), to improve sustainable production and tuber quality of potatoes.

In addition to root growth, the development of stem and leaves, which provides nourishment through photosynthesis (Mouzo et al. 2022), is vital for potato growth after germination. Positive and significant associations exist between the height of the tallest stem and the combined root and stem mass, as well as with tuber mass, suggesting that more robust plants usually yield more tubers (Fig. 2, left; Kacheyo et al. 2021).

Leaf number and leaf area index are strongly correlated, and these traits can be assessed through a single measurement (Liu et al. 2020). StHK proteins, including StHK3 and StHK4, act as CK receptors in potatoes and *StHK3 *gene is predominantly expressed in leaves (Lomin et al. 2018). MicroRNAs miR159, miR160, miR162, miR166, miR167, miR168, and miR171 are highly expressed in leaf and are crucial in determining potato leaf architecture (Yang et al. 2019). Recent study supports the involvement of miR160 in regulating potato leaf curvature by targeting StARF10/16/17 (Natarajan et al. 2020). Overexpressing the cytokinin-deactivating gene *CYTOKININ OXYDASE 2* (*CKX2*) exhibited an excessive growth of axillary shoots and branching (Pino et al. 2022). Understanding the regulatory mechanisms governing potato stem and leaf development could be key to improving potato crop productivity and quality.

#### Senescence in potato

Senescence refers to the gradual degradation or decline of cells, tissues, organs, and organisms with increasing age (Woo et al. 2019). In plants, pro-senescence serves as a self-protection mechanism under stress, but it also substantially limits crop yield potential, leading to economic losses. A negative correlation was observed between life cycle length and yield in C × E populations and specifically in the genotype CE3027 (Clot et al. 2023; Shi et al. 2024). However, varieties selected for the breeding program prioritized late senescence and early nodulation to maximize yields (Clot et al. 2023). Thus, understanding senescence regulation mechanisms is crucial for developing new varieties and enhancing potato yield.

Leaf senescence is a biological process that is coordinately regulated by multiple genes and is self-depleting, which involves highly complex genetic programs that are precisely controlled by multiple layers of regulation, including chromatin and transcriptional regulation, post-transcriptional regulation, translational regulation, and post-translational regulation (Guo et al. 2021). The Leaf Senescence Database (LSD, 4.0) contains 31,214 genes and 1,037 mutants from 86 different species, providing valuable resources for elucidating the regulatory mechanisms of leaf senescence (Cao et al. 2022). Leaf senescence is affected by age, plant hormones, and external factors such as light, temperature, and nutrient availability. The senescence process involves complex regulatory networks and interactions among multiple signaling pathways, shaped by both environmental factors and internal signals (Lim et al. 2007). Genetic and functional genomic studies of senescence-related genes have shed light on the regulatory mechanisms of potato senescence (Fig. 2, right), which are important for managing and potentially improving crop longevity and productivity.

### Hormones

Hormone content analysis in grafted potato plants demonstrated that early-maturing varieties exhibited higher levels of ABA and SA but lower levels of IAA compared with late-maturing varieties, indicating that elevated ABA and SA levels, along with decreased IAA levels, might contribute to the early physiological maturity of potato plants (Hui et al. 2022).

ABA insensitive 5 (ABI5), a basic leucine zipper (bZIP) transcription factor essential for ABA signaling, affects leaf yellowing and senescence when overexpressed in transgenic potatoes (Finkelstein and Lynch 2000; Zhu et al. 2020a, b). StABI5 like 1 (StABL1) interacts with StSP3D and StSP6A, creating new flowering and tuber formation complexes in a 14–3-3 protein-dependent manner, leading to early flowering and tuber formation, thereby shortening the growth cycle (Jing et al. 2022). Overexpression of *StUBA2a/b* in *Arabidopsis* triggers premature leaf senescence, with upregulation of genes related to SA signaling and biosynthesis in *StUBA2a/b*-OE plants (Na et al. 2015).

TCP transcription factor StAST1 (StABL1 and StSP6A-associated TCP protein 1) regulates the ripening syndrome by interacting with StSP6A and StABL1, which in turn reduces the formation of the nodulin-activated complex (aTAC). StAST1 regulates the ABA/GA activity through activation of *StGA20ox1*and interaction with StABL1 (Sun et al. 2024).

### Circadian clock

StCDF1, a DOF transcription factor, is regulated by circadian rhythms and interacts with StSP6A to influence tuber formation and plant life cycle. Removal of the C-terminal DOF3 region from StCDF1 alleles enhanced protein stability, promoting potato ripening (Kloosterman et al. 2013). In *Arabidopsis*, the FKF1-GI protein complex interacts with CDF1. The resulting complex suppresses the expression of *StCO1/2 *and alleviates the repression of StSP6A expression, thus promotes plant maturation and tuber development (Kloosterman et al. 2013). StCDF1 acts as a negative regulator of senescence by binding and suppressing *StORE1S02*, a homolog of *ORESARA1*, to shorten life cycle (Shi et al. 2024; Tai et al. 2024). StABL1 collaborates with florigen (StSP3D) and tuberigen (StSP6A) under both long-day and short-day conditions to advance potato maturity (Jing et al. 2022).

StABI5 is involved in chloroplast development and photosynthesis and is influenced by the circadian clock. Overexpressing *StABI5* in potato accelerates dark-induced leaf yellowing and senescence, revealing that darkness enhances the transcriptional activity of *StABI5*, triggering chlorophyll catabolism gene expression, leading to chlorophyll breakdown, and causing leaf yellowing and senescence (Zhu et al. 2020a, b). Red light treatment enhances APX activity more than other treatments, boosting antioxidant capacity and thus delaying leaf senescence (He et al. 2022).

### Stress

Salt stress is a major abiotic factor affecting senescence. Under salt stress, photosynthetic pigments, proteins and biomass of potato plants decrease, whereas ROS and MDA contents increase, as well as the soluble sugars, soluble proteins and proline contents due to the enhanced enzymatic activity of POD, SOD and CAT (Wang et al. 2023a, b). StHPT and St-gamma-TMT, key enzymes in tocopherol synthesis, are crucial for plant responses to salt stress. Mutants deficient in these enzymes demonstrated decreased tocopherol levels. When exposed to 400 mM NaCl, plants with reduced expression of *StHPT* and *St-gamma-TMT *exhibited delayed leaf senescence (Abbasi et al. 2007).

Drought stress reduces the photosynthetic efficiency of potato leaves (Obidiegwu et al. 2015). Darkness activates the transcriptional activity of *StABI5 *and induces the expression of chlorophyll catabolism related genes to accelerate the chlorophyll degradation, leading to leaf yellowing and senescence (Zhu et al. 2020a, b). *Verticillium dahliae*, a soil-borne fungus, triggers wilt, chlorosis, and premature plant senescence (Dung et al. 2014). Overexpressing *StPP2Ac2b* in potatoes increases susceptibility to pathogens, indicating that *StPP2Ac2b* positively influences pathogen-induced senescence. This effect might be linked to its role in controlling the spread of cell death after mechanical injury (Muñiz García et al. 2022).

### Secondary metabolites

Metabolomic analysis revealed that the early-maturing Z5 scion exhibited an increase in phenolic acids and flavonoids, whereas the late-maturing Z18 scion displayed a decrease in amino acids, phenolic acids, and alkaloids (Hui et al. 2022). Knockdown of GMPase in transgenic plants promotes a senescence phenotype, suggesting that GMPase affects senescence by increasing ascorbic acid content (Keller et al. 1999). StHPT, involved in tocopherol synthesis, is critical for senescence regulation; RNAi mutants of HPT showed faster leaf senescence and increased oxidative stress responses, highlighting the involvement of tocopherol in leaf senescence (Abbasi et al. 2007; Abbasi et al. 2009). Three phenolic acid compounds (1-caffeoylquinic acid, protocatechuic acid-4-glucoside, and trihydroxycinnamoylquinic acid) and a flavonoid glycoside (myricetin-O-glucoside-rhamnoside) inhibit the growth of precocious cultivars and associate with the precocious traits in potato (Hui et al. 2022).

### Sucrose, lipids and mRNA

Maturity is essential trait for breeders to select potato cultivars suitable to grow in different latitudes. *StSWEET10C* and *StSWEET11 *have been identified to be associated with early potato maturity through RNA-seq analysis and metabolomic assays (Hui et al. 2022). StORE1S02 promotes expression of *StSWEET11* and *SAG29/SWEET15*, increasing sugar transport and leading to senescence and yield loss in potatoes (Shi et al. 2024). However, whether leaf senescence is driven by sugar accumulation or deficiency remains to be clarified (van Doorn 2008). Although ethylene accelerates leaf senescence in potatoes, lysophosphatidylethanolamine (LPE) can delay ethylene-induced senescence and could mitigate the loss of apical dominance in micropropagated potato plantlets (Özgen et al. 2005). Additionally, long-distance transport signal molecules, mRNAs, are revealed to be involved in maturity of tetraploid cultivated potatoes by regulating transcript levels of related genes like *StCBF1*, *StCBF2*, *StMADS18 *(Hui et al. 2022).

### Flowering in potato

**Fig. 3:**
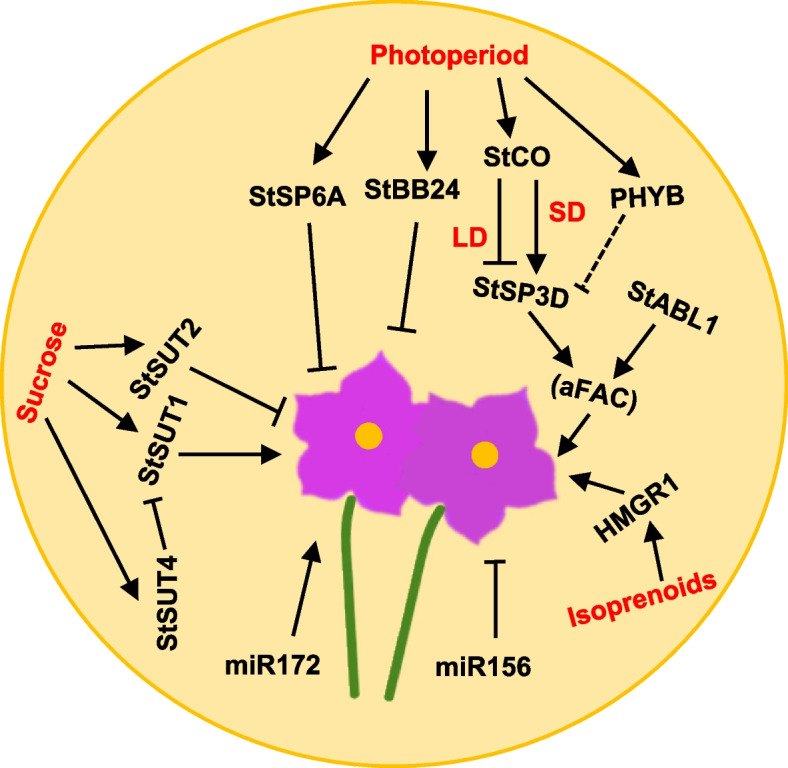
A diagram of the regulatory mechanism of potato flowering. Photoperiod, sucrose, microRNA, and isoprenoids play crucial roles in the regulation of potato flowering. StSP3D interacts with transcription factor StABL1, bridged by St14-3-3 s to form a flowering activation complex (aFAC) that promotes flowering. Under long day (LD) condition, *StCO* inhibits *StSP3D* expression, reducing the formation of aFAC and thereby inhibiting flowering. Conversely, under short day (SD) condition, *StCO* promotes the expression of *StSP3D*, which, in turn, promotes flowering. StSUT1, StSUT2, and StSUT4, sugar transporters

Plant flowering, marking the shift from vegetative to reproductive growth in the shoot apical meristem (SAM), involves dynamic changes in growth and development, cell division at the stem tip meristem, and subsequent morphological transformations. During flowering induction, the SAM transitions to an inflorescence meristem (IM), which then forms flower meristems (FMs) that produce flowers, fruits, and seeds. In *Arabidopsis*, the IM leads to the continuous growth of inflorescences (Shannon and Meekswagner 1991; Bradley et al. 1997; Larsson et al. 1998). At least six pathways regulating flowering, including the photoperiod, vernalization, gibberellin, autonomous, age-dependent, and temperature-sensitive pathways have been identified, as well as key genes such as *FLOWERING LOCUS T* (*FT*) and transcription factors such as CONSTANS (CO) (Fornara et al. 2010; Song et al. 2012; Song et al. 2014; Rosas et al. 2014). Recently, studies revealed the functional evolution and unique regulatory mechanisms of *FT *gene family in horticulture plants, indicating the promising applications of these genes as breeding targets (Wang et al. 2022a, b). Unlike *Arabidopsis*, potatoes are monoecious and homogamous, facilitating self-fertilization and hybridization. They can reproduce both sexually through flowering and asexually through tuberization. Flowering time in potatoes is affected by interactions between internal and external factors, including hormones, sugars, isoprenoids, and microRNAs, along with external cues such as photoperiod (Fig. 3).

#### Endogenous regulation

*Arabidopsis *FT is a key protein with strong flower-inducing activity (Wigge 2011). The homologous genes of FT have identified in tomatoes and potatoes, highlighting their importance in flowering signals (Kinoshita et al. 2011). In rice, the homologs Hd3a and RICE FLOWERING LOCUS T1 (RFT1) regulate flowering under short-day and long-day conditions, respectively (Komiya et al. 2008). In potatoes, flowering also relies on mobile FT-like proteins, with two homologs, StSP3D and StSP6A. StSP3D is primarily involved in flowering regulation, and its expression in leaves is controlled by photoperiod, whereas StSP6A is considered the main regulatory factor for tuber formation (Navarro et al. 2011). Additionally, three more FT-like genes (*StSP5G*, *StSP5G-like*, and *StSP3A*) have been found in potatoes (Xu et al. 2011). In tomatoes and rice, FT binds with FD and a 14–3-3 protein to form the so-called “flowering activation complex” (FAC), which regulates the expression of characteristic genes of the floral meristem tissue, such as *SOC1*, *AP1*, and *FUL (*Pnueli et al. 2001; Taoka et al. 2011). Interesting, FAC complexes similar to those observed in tomatoes and rice regulate the flowering process of potatoes (Almekinders and Wiersema 1991; González-Schain and Suarez-Lopez 2008).

StABL1 plays a role in the formation of alternative TACs (aTACs) and alternative FACs (aFACs) in potato, which are involved in flowering. Overexpressing *StABL1* results in the early initiation of flowering (Liu et al. 2019). StSP6A interacts with StABL1, bridged by St14-3-3 s, to form an FAC-like complex that promotes flowering (Teo et al. 2017). CO, a BBX (B-box type zinc finger) family member, enhances flowering in *Arabidopsis* in a photoperiod-sensitive manner. Heterologous expression of *CO* in potatoes inhibited flowering and stem block formation, indicating a different mechanism in potato flowering regulation (González-Schain and Suarez-Lopez 2008). The potato genome contains three CO-like genes (*StCOL1*/*StCOL2*/*StCOL3*), which are located in a tandem array on chromosome 2 (Abelenda et al. 2016). Silencing and overexpression of *StCO* in potato can lead to early flowering, which can be explained by the upregulation of potato FT homologous protein StSP3D. *StBBX24* is a clock control gene encoding a B-box protein located in the cytoplasmic and nuclear chromatin fractions and knockdown of *STBBX24* expression resulted in earlier flowering. By contrast, overexpression of *StBBX24 *did not produce flower buds when compared with wild-type plants (Kiełbowicz-Matuk et al.  2022), suggesting that the regulation of *StCO* expression is necessary to control flowering time in potato

#### Photoperiod

Photoperiod significantly influences potato flowering, with plants in regions with shorter daylight hours experiencing flowering earlier than those in areas with extended sunlight exposure. This timing ensures that potatoes flower at the optimal period for successful pollination and seed production. Key photoreceptors involved in photoperiod-induced flowering in potatoes include phytochrome A (StPHYA), phytochrome B (StPHYB), and phytochrome F (StPHYF). The silencing of *StPHYF* leads to an early flowering because it activates the florigen encoding gene *StSP3D* and other related genes. Additionally, transcriptional and microarray data identified many genes such as *StMADS1*, *StMADS13*, *StFDF1.1*, and *StSUT1*, which are potentially involved in both tuberization and flowering processes (Wang et al. 2023a, b).

Under short-day condition, accumulated StSP6A forms a TAC with StABL1 facilitated by St14-3–3 proteins, affecting GA metabolism, inhibiting GA activity at stolon tips, and promoting tuberization. Under both long-day and short-day conditions, StSP3D interacts with StABL1 through St14-3-3 s, creating an FAC-like complex that promotes flowering (Jing et al. 2022). Under short-day or long-day conditions, StCO enhances or represses *StSP3D *expression, promoting or inhibiting flowering, respectively (Navarro et al. 2011).

Daily light integral (DLI) significantly influences flowering time, with higher DLI advancing flower bud emergence by activating the *StSP3D* gene. Although *StSP3D *expression increased under high DLI, StSP3D-silenced lines also exhibited early flowering under these conditions, suggesting that high DLI-induced flowering is not solely dependent on the StSP3D pathway (Plantenga et al. 2019). High DLI might affect sucrose and *StTPS1* expression, potentially triggering *StSP3D* and accelerating flowering. Observations under various light conditions demonstrated that high DLI improves plant carbohydrate status, as determined by measuring sucrose and starch in leaves and stem tips, and *StTPS1 *expression in leaves. However, this does not indicate a direct link between carbohydrate status and the flowering process (Plantenga et al. 2019).

Flower development is expedited under a combination of low photoperiod and high light intensity, whereas high photoperiod or low light levels decelerate flowering. Genetic disruptions in *PHYF*, *StCOL1*, or *StSP5G *lead to swift flowering and tuberization under low-light conditions (Abelenda et al. 2016).

#### Hormones

GA4 binds tightly to its receptor GID1, forming a complex with the flowering inhibitor DELLA, which then undergoes ubiquitination and degradation via the 26S proteasome, mitigating the suppressive effect of DELLA on flowering (Wang et al. 2009). GA3 treatment also affected the reproductive growth of potato plants. For early maturing varieties with limited flowering capacity that do not bud, a 50% flowering rate can be induced. In varieties with strong flowering potential, GA3 can prompt early flowering and prolong the flowering duration. This effect may result from GA3’s ability to hinder tuberization, shift nutrient distribution, and facilitate inflorescence development (Wu et al. 2014).

#### Sucrose

Sugar plays a crucial role in the flowering control of various plants. The *sucrose transporter* (*SUT*) gene family is widely distributed in higher plants. Despite low homology among sucrose transport proteins from different species, they all fall under the category of proton sucrose co-transporters (Kuhn et al. 1997).

In potatoes, the role of carbohydrates in regulating flowering is less understood. Studies have indicated that *StSUT1 *predominantly facilitates sugar accumulation in tubers (Chandran et al. 2003). Research on *StSUT4 *suggested its involvement in flowering and tuber formation (Chincinska et al. 2008). RNA interference (RNAi) targeting *StSUT4* during potato flowering reduced *StSUT1 *expression, increased plant sucrose levels and promoted early flowering and higher tuber yield (Chincinska et al. 2008). StSUT2-RNAi affects flowering time and tuber yield without affecting carbohydrate storage in leaves and tubers, possibly influencing cell wall component metabolism (Gong et al. 2023).

MiR172 is involved in phloem movement and sugar-dependent signaling for flower and tuber initiation in potato plants, illustrating the link between solute transport and the onset of flowering and tuberization. The expression of mature miR172 in wild-type and *StSUT4*-silenced potato plants is dependent on sucrose signaling (Garg et al. 2021).

#### Isoprenoids and microRNAs

Isoprenoids, a large group of plant natural products, play major roles in plant growth and development. Overexpression of *HMGR1 *in plants leads to increased levels of sterols, steroidal glycoalkaloids (SGAs), and plastidial isoprenoids, resulting in early flowering, greater stem height, more biomass, and higher total tuber weight (Moehninsi et al. 2020). Overexpressing miR156, which may act as a graft-transmissible signal, leads to reduced tuber yield and delayed flowering (Bhogale et al. 2014). Conversely, miR172 enhances flowering and initiates tuberization even under noninductive conditions (Martin et al. 2009).

Further research is required to fully elucidate the specific regulatory mechanisms and pathways of the flowering process in potatoes, which is essential for understanding potato reproductive development and could contribute to advancements in potato breeding.

### Potato tuberization

**Fig. 4:**
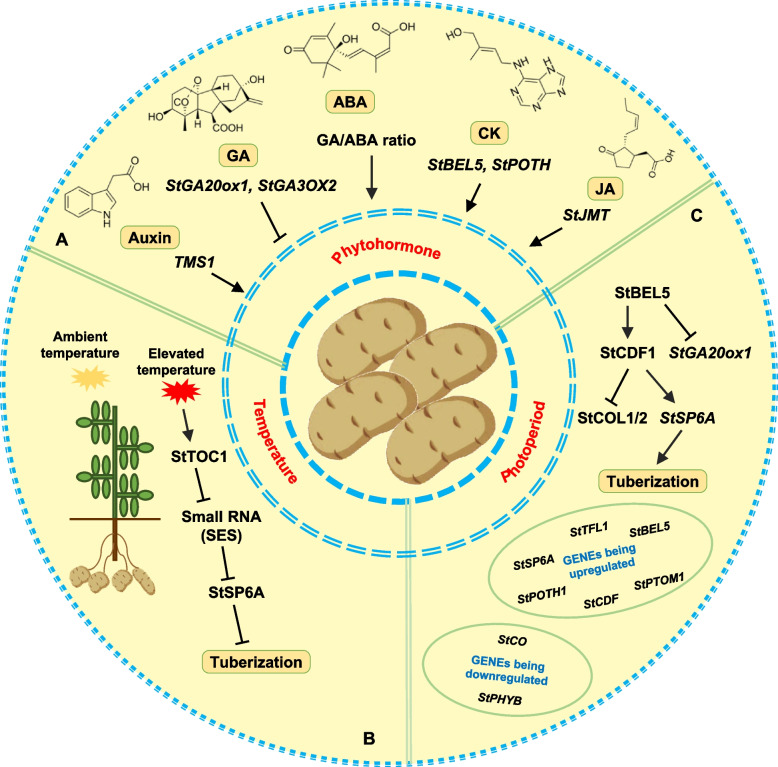
Regulatory factors involved in potato tuberization. **A** Auxin, ABA, CK, and JA promote, whereas GA inhibits, tuberization. **B** Under high temperature, *StTOC1* inhibits the expression of *StSP6A* by acting on microRNA. **C** StSP6A and StBEL5 produced in leaves are transported to the stolon stem tip as a movement signal to induce tuberization, and StCOL1/2 inhibits tuberization by inhibiting the accumulation of StSP6A and StBEL5 mRNAs in leaves

As the storage and harvesting organ of potatoes, tubers are a vital source of starch, protein, antioxidants, and vitamins. Tubers form in the near apical region of underground stolons (Ewing and Struik 1992) and the formation process is categorized into four stages: stolonogenesis, tuber induction and initiation, tuber enlargement, and maturation (Viola et al. 2001). The development of potato tubers is co-regulated by various internal factors and external conditions, which activate multiple signal transduction pathways, exhibiting intricate upstream and downstream relationships and interactions (Fig. 4).

#### Hormones

##### Auxin

Auxin exhibits an increase in its levels prior to and during tuber growth, and plays an important role in tuberization (Roumeliotis et al. 2012). Studies indicate that auxin positively affects tuberization in a concentration-dependent manner, with IAA content in the sub-apical stolon region rising by 45% during apical expansion (Koda and Okazawa 1983). Applying 2 to 10 mg/L IAA externally promoted early tuberization by regulating sucrose accumulation at the stolon tips (Wang et al. 2018; Zhang et al. 2005), highlighting the link between auxin and tuberization. DR5 promoter-driven GUS expression and staining revealed a marked increase in auxin levels in stolons before tuberization, remaining high throughout tuber growth, emphasizing auxin's crucial role in this phase (Roumeliotis et al. 2012). The upregulation of the auxin synthesis gene *TMS1*, driven by a tuber-specific promoter (B33 promoter), during tuberization corresponds with increased auxin levels and reduced carbohydrate and photoperiod dependence for tuber enlargement in vitro (Kolachevskaya et al. 2015). This suggests the positive effect of auxin on potato tuber enlargement, indicating its potential to boost potato yield by augmenting auxin levels.

Analysis of the potato genome revealed numerous genes essential for auxin signaling comparable to those in *Arabidopsis*. For example, potato has five receptor genes (six in *Arabidopsis*) (Kolachevskaya et al. 2017), 22 ARF genes (23 in *Arabidopsis*), and 25 *AUX/IAA* genes (29 in *Arabidopsis*) (Kumar et al. 2015). The auxin-related genes are differentially expressed in the early stages of tuber development. A study demonstrated the involvement of *StIAA* in tuber development. When 4-week-old plants were treated with IAA (10 μM) for 3 h, 12 *StIAA *genes were highly expressed in stolons, most of which were auxin-sensitive (Gao et al. 2016). StIAA1 gene is accumulated in potato tubers after fungal infection and injury, and the expression level of *StIAA1* mRNA in potato leaves significantly increased under IAA treatment (Zanetti et al. 2003). Silencing the expression of the *StIAA2 *gene led to increased plant height, lower petiole attachment, and extreme curvature of stem tip leaf primordia. This was accompanied by altered expression of several downstream genes, including other Aux/IAA family members (Kloosterman et al. 2006).

During the early stages of tuber development, auxin levels in stolons increased, accompanied by the upregulated expression of two PIN family genes, suggesting the substantial role of auxin transport in potato tuberization (Kloosterman et al. 2008). In sequenced potatoes, 10 PIN family genes were identified, with *StPIN3* and *StPIN4* predominantly active in stolons and *StPIN1* and *StPIN4 *mainly active in young tubers (Roumeliotis et al. 2013a, b). The expression of *GUS* genes driven by StPIN2/4 promoters was observed in the vascular bundles during the initial tuber stage and in parenchyma cells during rapid tuber growth, highlighting the crucial role of *StPIN *genes in auxin distribution during early tuber development (Roumeliotis et al. 2013a, 2013b).

##### GAs

High endogenous GA levels associated with the uninduced state of potato, inhibiting tuberization and encouraging stolon elongation (Railton and Wareing 1973; Xu et al. 1998). The primary bioactive GAs, GA1 and GA3, decrease rapidly before tuberization after being elevated during stolon growth (Malkawi et al. 2007). Tuberization can be induced by applying GA biosynthesis inhibitors, with high GA levels at stolon tips favoring meristem elongation and lower GA levels needed for tuberization initiation.

GA 20-oxidase (GA20ox) is crucial for synthesizing active GAs (Hedden and Thomas 2012). *StGA20ox1 *overexpression resulted in delayed tuber development (Roumeliotis et al. 2013a, b). Similarly, StGA2ox1 is essential for tuberization, with its expression increasing in early tuber development stages, especially in the subapical region of stolons and growing tubers (Kloosterman et al. 2007). The activity of StGA2ox1 at tuber initiation likely facilitates normal tuber growth by altering GA levels. Moreover, *StGA3ox2 *overexpression delays tuber formation, whereas StGA3ox2 RNAi lines produce more tubers (Carrera et al. 2000; Jackson et al. 2000; Bou Torrent et al. 2011; Roumeliotis et al. 2013a, b).

##### ABA

The endogenous level of ABA increases during tuber induction and declines during tuberization. Although the role of ABA in tuberization is not fully understood, it is hypothesized to stimulate tuberization by counteracting the inhibitory effects of GA (Grandellis et al. 2016). Initially, as tuberization begins, the ABA/GA ratio markedly increases. When the tuber swells, ABA levels rise and GA levels drop. However, tuber formation is substantially affected by the GA/ABA ratio and not by the absolute hormone concentration (Macháčková et al. 1998).

##### CK

CK is pivotal in regulating potato tuberization, affecting cell division and proliferation. Early tuber growth exhibited a positive correlation between cell proliferation rate and the levels of auxin and CK (Raspor et al. 2020a). Endogenous CK peaks during tuber formation, and studies have indicated that applying exogenous CK (3 mg/L) or upregulating CK synthesis genes in tubers enhances tuberization (Cheng et al. 2020; Koda and Okazawa 1983; McGrady et al. 1986; Raspor et al. 2020a, b). However, overexpressing genes encoding CK oxidase, an enzyme that inactivates CK, results in fewer tubers, and overexpressing the *IPT *gene reduces tuber yield, complicating the determination of the precise role of CK in tuberization (Hartmann et al. 2011). During tuber development, BEL and POTH signaling factors modulate CK synthesis. Following CK synthesis, it suppresses the expression of *POTM1 *through receptor histidine kinases (StHKs), fostering cell division in the meristem and encouraging starch accumulation in the tubers (Lomin et al. 2018; Mariana et al. 2006).

##### JA

JA enhances potato tuber formation by counteracting the effects of GA (Aksenova et al. 2012). Initial studies have identified 12-OH-JA (tuberonic acid, TA) and its glucoside (TAG) as key substances promoting tuber formation and development (Yoshihara et al. 1989). TA and TAG, synthesized in leaves and transported to stolon tips, increase JA levels there, inducing stolon swelling (Koda et al. 1991; Takahashi et al. 1994). Further research showed increased jasmine content in the newly formed potato tuber cortex (Abdala et al. 1996). JA and TA levels in tubers and adjacent stolons decrease during tuberization (Abdala et al. 2002; Cenzano et al. 2005). Enhancing tuber JA content through exogenous application or gene overexpression promotes tuberization (Abe et al. 1990; Cenzano et al. 2007; Koda et al. 1991; Sohn et al. 2011), illustrating the role of JA in tuber formation and development. Overexpression of the transcription inhibitor StJAZ1 of JA signaling pathway can inhibit the initiation and enlargement of the top tubers of creeping stems, leading to a decrease in yield (Begum et al. 2022).

Despite the promotive effects of JA on tuberization, its specific role remains debated due to varying experimental JA concentrations (Ruiz del Castillo et al. 2010). Low JA concentrations (0.03 to 1 μM) thicken potato meristems and boost stem, leaf, and root growth (Castro et al. 1999; Cenzano et al. 2003; Ruiz del Castillo et al. 2010), whereas high concentrations (> 10 μM) may not enhance and can even inhibit tuber growth (Ravnikar and Gogala 1990; Ravnikar et al. 1992; Ruiz del Castillo et al. 2010). Applying JA at optimal concentrations (1 to 10 μM) is crucial for promoting tuber cell enlargement and formation (Pelacho and Mingo-Castel 1991; Pruski et al. 2002; Sarkar et al. 2006).

#### Temperature

High temperature inhibits tuberization in potatoes (Hijmans 2003), with this negative effect being consistent across different potato varieties (Van Dam et al. 1996; Lehretz et al. 2019; Singh et al. 2015). StSP6A plays a role in temperature-dependent tuberization, with its expression decreasing under high-temperature conditions (Hancock et al. 2014; Lehretz et al. 2019; Morris et al. 2019). The potato gene *StTOC1*, analogous to *Arabidopsis TOC1*, acts as a heat-responsive transcriptional regulator that suppresses *StSP6A *expression (Hancock et al. 2014; Morris et al. 2019). Silencing *StTOC1* increases tuber yield and StSP6A transcription levels. The *StSP6A* promoter contains a TOC1 recognition motif, suggesting the direct interaction of *StTOC1* with the *StSP6A *promoter (Morris et al. 2019). Additionally, a microRNA named SUPPRESSING EXPRESSION OF SP6A (SES) targets and negatively regulates the *StSP6A* transcription. Overexpressing *SES* reduced *StSP6A* transcription, but not when targeting codon-optimized *StSP6A* ^*coop*^. Overexpressing *StSP6A*^*coop*^enhanced potato tuber yield under high temperatures (Lehretz et al. 2019).

Recent research has highlighted the significance of posttranscriptional regulation in the early phase of transcription and transcriptional regulation as a key late-stage factor that suppresses *StSP6A* expression in leaves under high temperatures. Overexpressing *StSP6A *in leaves counteracted the suppression of tuber formation caused by high temperatures, but it did not mitigate the later-stage decline in tuber yield, likely due to inhibited sugar transport. Transcriptome analysis identified potential regulators involved in the thermal response of tuberization at various stages, suggesting that potatoes employ distinct molecular mechanisms over time to effectively manage tuber development at elevated temperatures (Park et al. 2022). GA biosynthesis inhibitor chloroethyltrimethylammonium chloride (CCM) could counteract the adverse effects of high temperature on tuber formation when applied to plants or stem segments (Menzel 1983). By overexpressing *StGA2ox1 *in potatoes, the level of bioactive GA decreased, promoting tuber formation (Dong et al. 2017; Shi et al. 2016). Overexpression of*StSP6A* also triggered *StGA2ox1 *expression (Kloosterman et al. 2007; Navarro et al. 2011). However, the molecular mechanisms within the temperature-responsive components of the GA signaling pathway and the exact relationship between StSP6A and GA remain unclear.

#### Photoperiod

##### Phytochrome

The key photoreceptors associated with photoperiod-induced tuberization in potatoes are StPHYA, StPHYB, and StPHYF as described above. StPHYA detects light signals under far-red light condition, contributing to the reorganization of potato biological rhythms and tuber formation. StPHYB, accumulating stably in green leaves, acts as a long-distance signaling molecule, responding to photoperiod changes under red light. StPHYF and StPHYB potentially regulate potato tuberization through heterodimer formation, suppressing tuber formation under non-inductive long-day conditions. Additionally, the blue light receptor FLAVIN-BINDING KELCH REPEAT F-BOX PROTEIN 1 (FKF1) in potatoes forms a complex (StFKF1/StGI) with the circadian clock core regulator GIGANTEA (GI), utilizing blue light to gauge day length (Zhou et al. 2019).

### Constans (CO) and Cycling Dof Factor 1 (CDF1)

Transcription factor CO, crucial for regulating flowering and tuberization, is tightly controlled by circadian rhythms. Potatoes have two CO variants (StCO1 and StCO2), also known as StCOL1/2, which negatively influence tuberization (Navarro et al. 2011). *StCO *overexpression delays tuberization, with its inhibitory impact on tuberization transmissible via grafting (González-Schain et al. 2012). The DOF (DNA-binding with one finger) gene family, unique to plants, modulates the expression of various genes during growth and development. The CDF1 (StCDF1) protein, part of the DOF family in potatoes, interacts with StGI1 and StFKF1, downstream of clock components StGI1 and StFKF1. The StFKF1/StGI complex influences StCDF1 stability, which in turn regulates *StCOL1/2 *expression, making StCDF1 a vital player in the circadian clock's regulation of StCO (Kloosterman et al. 2013; Sawa et al. 2007).

### Flowering locust (FT) homologous genes

FT proteins, produced in leaves and transported via phloem, are recognized as signaling molecules that trigger flowering, with CO proteins serving as transcription factors regulating *FT *expression (González-Schain et al. 2012). Grafting studies in tobacco and potato have demonstrated that florigenin, or tuberogenin, acts as a mobile signal for both flowering and tuberization (Abelenda et al. 2014), implying that these processes might share a common signaling molecule. Potatoes have four FT-like genes: *StSP6A*, *StSP5G*, *StTFL1*, and *StSP3D*. StSP6A is confirmed to be a mobile signal from leaves to stolon tips under long-day conditions, inducing tuberization. In photoperiod-sensitive varieties, limited sunlight triggers StSP6A mRNA accumulation in leaves and stolons, enhancing tuberization (Navarro et al. 2011; González-Schain et al. 2012). Inducible promoter-driven *StSP6A* expression also activates tuber marker genes, with higher expression in early-maturing and lower in late-maturing varieties, indicating a link between *StSP6A* expression and tuber maturation, though the transcriptional regulation mechanism remains unclear.

Under long-day conditions, StPHYB detects light signals and forms a complex with E3-ubiquitination ligase FKF1 and nuclear protein GI, which then regulates StCDF1, influencing *StCOL1/2 *expression and consequently inhibiting StSP6A and tuberization (Sawa et al. 2007). The impact of StCOL1/2 on tuber induction, confirmed through grafting, suggests its remote regulatory role in signaling molecule gene expression, aligning with StCDF1's modulation of StCOL1/2 and StSP6A activity. StCOL1/2's suppression of StSP6A is also affected by StSP5G (Abelenda et al. 2016; Zhou et al. 2019). Additionally, the FT-like gene StTFL1 maintains high mRNA levels in stolons during tuber induction and early development, with *StTFL1 *overexpression increasing tuber numbers (Guo et al. 2010), highlighting its role in tuber induction and growth. While StSP3D is known to influence flowering (Navarro et al. 2011), its effect on tuberization remains unreported.

### StBEL5 and POTH1

StBEL5 acts as a positive regulator of tuberization, with its mRNA produced in leaves and transported via the phloem to underground stolon tips (Banerjee et al. 2007). *StBEL5* expression in leaves is triggered by low levels of blue and red light, but remains unaffected by sunlight duration. *StBEL5 *overexpression leads to early tuberization, while RNAi-mediated StBEL5 suppression notably decreases tuber yield (Sharma et al. 2016). The StBEL5 protein triggers various tuber-forming genes, including StSP6A and StCDF1 (Abelenda et al. 2016; Sharma et al. 2016). During tuberization induction, StSP6A and StBEL5, produced in leaves, are transported to the stolon's tip, while StCOL1/2 counters tuberization by reducing StSP6A and StBEL5 transcriptions in leaves (Hannappel et al. 2017).

*POTH1* gene expression in potatoes is light-induced, with POTH1 mRNA moving to the stolon tip to aid tuberization. Research indicates POTH1 and StBEL5 together suppress the potato gibberellin oxidase gene *StGA20ox1*, converting active GA1 and GA4 into inactive GA8 and GA34, thus influencing GA synthesis during tuberization (Chen et al. 2004). The StBEL5-POTH1 complex might also elevate cytokinin levels, suggesting StBEL5 and POTH1 collaboratively influence tuber formation by regulating hormone levels at the stolon tip (Chen et al. 2004).

Development of potato tubers is also regulated by post-transcriptional regulation. N6-Methyladenosine (m6A) is the most abundant internal chemical modification in eukaryotic mRNA, which widely involved in the regulation of plant growth and development, plant–microbe interactions, plant-environment interactions, and crop trait improvement through regulating RNA stability, alternative polyadenylation, chromatin state, translation, and miRNA events (Tang et al. 2023; Zhou et al. 2022). Recently, it was demonstrated that the human FTO-mediated plant m6A removal in potato promoted tuber enlargement and caused ~ 50% increases in yield and biomass in field trials, demonstrating that modulation of plant RNA m6A methylation is a promising strategy to improve yield (Yu et al. 2021).

In conclusion, potato tuberization involves a complex interplay of external environmental factors, endogenous hormones, and signaling molecules that activate or repress specific genes, manage biochemical pathways, and direct assimilate distribution, causing stolon enlargement. Future research should aim to dissect the roles of key genes, metabolic pathways, and signaling molecule interactions in tuberization, offering insights for developing high-yield and high-quality potato cultivars via precise molecular breeding.

### Accumulation of storage compounds

Potatoes are rich in various nutrients, serving as a significant source of balanced nutrition in human diets. Tubers are primarily composed of carbohydrates, which constitute about 70%–85% of the dry matter, mostly as starch; they also have low lipid and protein contents. Potatoes are particularly high in vitamin C, contributing to the antioxidant activity, and are a good source of various B vitamins. The potato tuber skin is rich in dietary fiber and potassium.

#### Starch biosynthesis and degradation

As the primary stored carbohydrate in potato tubers, potato starch consists of two glucose polymers: amylose (about 18%–21% of starch content) and amylopectin (about 79%–82% of starch content) (Zeeman et al. 2010), which are stored in semicrystalline, insoluble granules with growth rings, predominantly found in plastids and known as starch granules (Buléon et al.  1998). The formation of the growth rings of starch granules is influenced by the structure of starch polymers (Pilling and Smith 2003). In addition, potato starches harvested at different growth stages show significant variations in structure and physicochemical properties (Yang et al. 2021b).

Potato starch biosynthesis and degradation occur in distinct organelles based on the tissue type: chloroplasts in autotrophic organs and amyloplasts in heterotrophic organs, respectively (Lloyd and Kossmann 2015). Transient storage starches in source tissues meet basic energy demands, such as those required for diurnal cycles and biological processes (Orzechowski 2008). In photosynthetically active leaf tissue, starch synthesis starts with the conversion of photosynthesis product fructose 6-phosphate (F6P) to ADP-glucose through a series of enzymatic reactions catalyzed by phosphoglucoisomerase (PGI), phosphoglucomutase (PGM), and ADP-glucose pyrophosphorylase (AGPase) in the chloroplast (Sonnewald and Kossmann 2013). While in the sink organ tuber, sucrose unloaded from the phloem is hydrolyzed into UDP-glucose and fructose by sucrose synthase (SuSy) in the cytoplasm (Bahaji et al. 2014). In amyloplasts, G6P is converted back to G1P by plastid PGM, then ADP-glucose is generated by AGPase, serving as the substrate for starch biosynthesis (Van Harsselaar et al. 2017).

Long chain linear and branched starch molecules are formed by the action of starch synthases (SSs), granular-bound starch synthases (GBSSs), starch branching enzymes (SBEs) using ADP-glucose, and debranching enzymes (DBEs) that trim excess or improper branch points (Zeeman et al. 2010; Bahaji et al. 2014). Soluble SS enzymes of potato: SSI, SSII, and SSIII, are crucial in amylopectin synthesis by elongating α-glucan chains (Ball and Morell 2003; Patron and Keeling 2005). These enzymes extend short, medium, and long chain starches, respectively (Brust et al. 2013), with SSIII contributing to about 80% of the soluble starch synthase activity in tubers (Tomlinson and Denyer, 2003; Cuesta-Seijo et al. 2016). The fourth isoform, SSIV, is thought to regulate the initiation of starch granules (Pfister and Zeeman 2016). Notably, there is species- and organ-specific variation in these enzymes' activities (Bertoft 2017). Additionally, the insoluble enzyme granule-bound starch synthase (GBSS) primarily synthesizes amylose (Denyer et al. 2001). In *Arabidopsis*, the PROTEIN TARGETING TO STARCH (PTST) interacts with GBSSI's catalytic domain to localize it to the starch granule (Seung et al. 2015). SBEs facilitate branched starch synthesis and reducing the activity of SBEI and SBEII results in up to 56% increase in amylose content, reduced branched starch, and an increase in long-chain starch molecules (Tetlow and Emes 2014).

In starch degradation, glucose and maltose are the two primary final products, involving reversible glucan phosphorylation and hydrolysis pathways (Lorberth et al. 1998; Ritte et al. 2002; Ritte et al. 2006). Phosphoglucan, water dikinase (PWD), contrasts with glucan, water dikinase (GWD) by phosphorylating the C3 position of starch residues, whereas GWD targets the C6 position (Baunsgaard et al. 2005; Hejazi et al. 2010). Amylopectin's crystalline layers are progressively degraded through phosphorylation and dephosphorylation by phosphorylases and phosphatases, respectively, exposing new layers for degradation. Debranching enzyme (DBE) is crucial for hydrolyzing α-1,6 branch points in amylopectin (Delatte et al. 2006) and DBEs are categorized into ISAs (ISA1, ISA2, ISA3) and LDA based on amino acid sequence and substrate specificity (Møller et al. 2016). ISA1 and ISA2 are directly involved in amylopectin synthesis, while ISA3 and LDA participate in starch degradation (Delatte et al. 2006). GWD, PWD, SEX4, and DBE contribute to breaking down amylopectin into glucans.

**Fig. 5:**
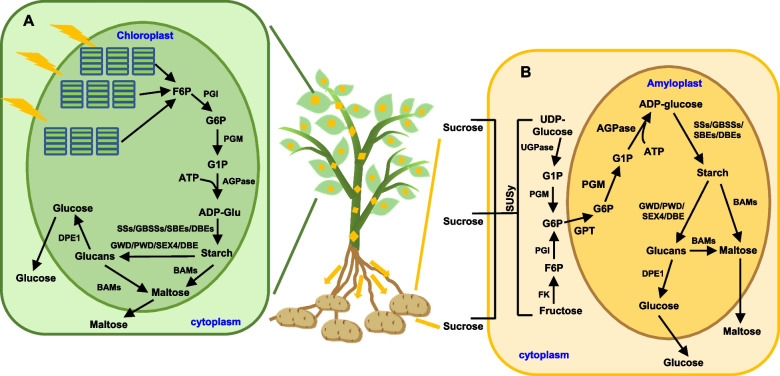
Starch metabolism pathway in potato source (**A**) and sink tissues (**B**). **A** F6P produced by photosynthesis in chloroplasts generates substrates for starch synthesis, ADP-Glu, through the enzymatic reactions of PGI, PGM and AGPase. SSs, GBSSs, SBEs, and DBEs continuously elongate into amylose and amylopectin. GWD, PWD, and SEX4 phosphorylate and dephosphorylate starch particles, increasing the water solubility and promoting starch degradation. DBEs are necessary for the degradation of branched starch. Amylose is hydrolyzed by BAMs to produce maltose, and then transported to the cytoplasm for metabolic utilization. **B** The substrate ADP-Glu for starch synthesis is synthesized in amyloplast. Sucrose derived from the unloading of the phloem is hydrolyzed by SUSy in cytoplasm to produce UDP-Glu and Fructose, which are then catalyzed by UGPase/PGM and FK/PGI to produce G6P, respectively. With the help of GPT, G6P enters the amyloplast to generate ADP-Glu by PGM and AGPase. Subsequent synthesis and degradation are similar to those in the above-ground parts. The yellow diamond represents sucrose, and arrows indicate the direction of sucrose transport

For amylose, characterized by linear chains, beta-amylase (BAM) releases maltose from the non-reducing ends of exposed chains (Scheidig et al. 2002). The shortest chains BAM can identify are maltotetraose, and disproportionating enzyme (DPE) transfers maltosyl groups from maltotriose, formed during starch degradation, to other glucans, producing free glucose and longer glucans for BAM metabolism (Asatsuma et al. 2005). Finally, glucose and maltose are transported from plastids to the cytoplasm for metabolism, facilitated by specific transport proteins (Fig. 5, Thorens and Mueckler 2010; Niittylä et al. 2004).

Beyond starch, potatoes contain notable levels of small sugars like glucose, fructose, and sucrose, which typically diminish as tubers develop (Navarre et al. 2013). These sugars contribute to intracellular energy metabolism, serving as ATP carriers and interacting with starch synthesis and degradation.

#### Starch metabolism regulation

The content of starch and the ratio of amylopectin to amylose significantly influence the starch's final structure, growth, development, and physicochemical properties, such as gelatinization, which are crucial for starch processing and applications. Sucrose in storage tissues originates from phloem unloading, and studies indicate that the relationship between sucrose supply and diurnal metabolic fluctuations is not directly proportional to starch synthesis in tubers. Instead, enzyme activities in the starch synthesis pathway are key determinants of tuber development (Zrenner et al. 1995). Enhancing SuSy activity in tubers boosts levels and total yields of starch, ADP-glucose, and UDP-glucose (Baroja-Fernández et al. 2009). CRISPR/Cas9-induced mutations in branching enzyme (SBE) genes in tetraploid potatoes increase the amylose content and elongate branched starch chains (Zhao et al. 2021); similarly, *SBEII *overexpression enhances short-chain branching in starch, altering the physicochemical properties of potato tuber starch, aiding in its processing and production (Brummell et al. 2015). CRISPR/Cas9-mediated *GBSS1 *mutations result in reduced or completely eliminated amylose in some transgenic tubers (Toinga-Villafuerte et al. 2022). Somaclonal variant Ros 119, identified through breeding, exhibited a 42 and 61% increase in fresh and dry tuber weights, respectively, due to the upregulation of six starch synthesis genes: *AGPase*, *GBSSI*, *SBEI*, *SBEII*, *SSII*, and *SSIII *(Adly et al. 2023).

Proteins influencing starch content and properties also play significant roles in starch metabolism. The knockout of *StTST1 *enhances tuber starch synthesis and curtails degradation under cold storage (Liu et al. 2023a, b). Disrupting the expression of the vacuolar membrane glucose transporter *StTST3.1*leads to cytoplasmic glucose accumulation, which hinders starch degradation and results in maltose buildup, influencing transitory starch turnover and potato plant growth (Liu et al. 2023a, b). The ectopic expression of the *AtCDF1 *transcription factor boosts starch and amino acid content, as well as yield in tubers under field conditions (Carrillo et al. 2023). Overexpressing *StDREB1 *yields tubers with greater weight than wild-type plants, affecting tuber quality in terms of dry matter, starch content, and reducing sugars in both greenhouse and field conditions (Chiab et al. 2021). In developing tubers, SnRK1 and HXK1 stimulate post-translational redox activation of AGPase via distinct sugar signaling pathways, modulating starch synthesis rates and ensuring suitable sucrose availability (Tiessen et al. 2003). In addition, starch synthesis in potato tubers is governed by redox modification of AGPase, clarifying the link between starch synthesis and sucrose supply (Tiessen et al. 2002). Thus, the starch metabolism regulatory network encompasses targeted metabolic enzyme gene edits, indirect signaling pathways tied to environmental and developmental factors, and feedback mechanisms involving small molecule sugars and starch, likely integral to sugar signaling.

#### Other storage substances

Potato tubers contain small amounts of other nutrients beneficial to human health. Potato proteins, which make up 1–2% of tuber fresh weight, with patatins accounting for 40% of this storage protein (Shewry 2003). Potato protein comprises 19 amino acids, with asparagine being the most prevalent (Zhu et al. 2010), making potatoes one of the richest protein sources among root and tuber crops (FAO, 2008). Transgenic potatoes overexpressing the seed protein AmA1 exhibited a 60% boost in total protein content and significant increases in total biomass and several essential amino acids (Chakraborty et al. 2010). Lipids constitute about 0.1% of potato fresh weight (Galliard 1973), with up to 90% of fatty acids being linolenic and linoleic acids, essential unsaturated fatty acids for human nutrition, alongside palmitic acid (Ramadan and Oraby 2016). Other bioactive lipid compounds like glycolipids, phospholipids, sterols, tocols, and carotenoids serve as cell membrane components or antioxidants. Though the total lipid content is low, overexpressing *WRI1*, *DGAT1*, and *OLEOSIN *have significantly increased TAG accumulation, phospholipids, and galactolipids, albeit with a reduction in starch content and an increase in soluble sugars (Liu et al. 2017). Upregulation of the 14–3-3 protein in transgenic tubers can augment total lipid content by up to 69% (Prescha et al. 2001).

Compared with other food crops, potatoes have the most comprehensive vitamin content, with vitamin C being particularly abundant as well as several B vitamins (Augustin 1975; Brown 2005; Zaheer and Akhtar 2016), and critical minerals in human diets (Brown 2008). Research on functional genes related to vitamin content is scarce, with studies focusing on optimizing vitamin retention across various cooking methods (Furrer et al. 2018). Dietary fiber, mainly found in potato skins, comprises about 1–2% of the potato's dry weight (Kolasa 1993). Potassium is the most abundant mineral in potatoes, followed by phosphorus and calcium, with fresh weight (FW) ratios of 564, 30–60, and 6–18 mg/g, respectively (Buckenhuskes 2005).

Similar to dietary fiber, potassium is predominantly found in the potato's flesh, making whole potatoes, with their skins, nutritionally superior (Camire et al. 2009). Secondary metabolites and small molecule substances in potatoes include glycoside alkaloids, protease inhibitors, lectins, phenolic compounds, chlorophyll, and anthocyanins. Notably, glycoside alkaloids, natural toxins produced during tuber germination, serve as a defense mechanism against various threats and are primarily concentrated in the potato skin, especially in the sprouting areas (Furrer et al. 2018). Despite their significance, research on these valuable, albeit small in quantity, storage materials in potato tubers remains limited. Future efforts should focus on leveraging these nutrients to enhance the potato's status as a "natural nutrition plant".

Overall, potatoes are favored for their high energy and low-fat profile, appealing to health and fitness enthusiasts. Their diverse nutrient content can satisfy nearly all human nutritional requirements, enhancing and breeding potato varieties rich in nutrients is becoming increasingly crucial.

### Biotic and abiotic stress

#### Biotic stress

**Fig. 6:**
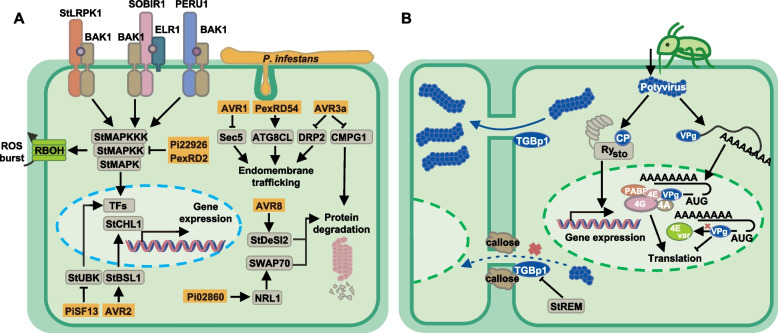
Potato immunity responses against *P. infestans* (**A**) and potyvirus (**B**). **A** Ligands secreted by *P. infestans* bind to pattern recognition receptors (PRRs), triggering immune complex formation and downstream kinase phosphorylation. StMAPKs cascades are phosphorylated to activate related transcriptional factors and induce ROS burst. To combat PTI immunity, *P. infestans* secrete various AVRs to aim specific protein targets. For endomembrane trafficking: AVR1 to Sec5, PexRD54 to ATG8CL, AVR3a to DRP2. For protein degradation: AVR3a to CMPG1, AVR8 to StDeSI2, and Pi02860 to NRL1, SWAP70. For StMAPK kinase activity, both PexRD2 and Pi22926 are involved. AVR2 interacts with StBSL1 to induce StCHL1. PiSF13 targets StUBK to reduce immunity responses. **B** Once potyvirus enters the plant cell through insect media, the coat protein (CP) is recognized and bound by a TIR-NLR immune receptor (e.g. Ry_sto_), and downstream responses are induced. The VPg binds to the initiation factor eIF4E to initiate viral transcription. eIF4E variants can escape interaction and improve resistance. Viruses spread through plasmodesmata with the help of TGBp1. Under virus invasion, StREM promotes callose deposition and inhibit TGBp1 function

As potato cultivation expands and distribution regions diversify, coupled with global environmental changes and other factors, potatoes are increasingly facing various biological stresses. Potato cultivation is affected by three major types of biological diseases: early blight and late blight caused by oomycetes (Adolf et al. 2020), bacterial wilt and ring rot caused by bacteria (Charkowski et al. 2020), and infections by various viruses and viroids (Kreuze et al. 2020). Understanding how diseases work and their molecular mechanisms is vital for controlling them efficiently and potato late blight and potato Y virus have been extensively examined (Fig. 6).

#### Potato late blight

Potato late blight, caused by the oomycete pathogen *Phytophthora infestans*, led to the Great Famine in Ireland and remains a major global threat to potato crops (Kamoun et al. 2015). In addition to infections, potatoes experience secondary infections after initial infection, leading to tuber rot and rendering them unusable (Dong and Zhou 2022). The economic impact of late blight diseases is substantial, with annual losses estimated between 3 and 10 billion USD (Kamoun et al. 2015).*P. infestans*, as one of the most economically and scientifically significant plant pathogenic oomycetes, has been the subject of extensive molecular mechanism studies (Kamoun et al. 2015).

The relationship between potatoes and *P. infestans *aligns with the classic “zig-zag” model of plant immunity (Jones and Dangl 2006), which describes how plants use two types of immune receptors, those on the plasma membrane and those within the cytoplasm, to detect pathogens such as bacteria and oomycetes, initiating immune responses. *P. infestans*secretes structurally conserved elicitor proteins called elicitins (ELIs) (Jiang et al. 2006), which bind to sterols, aiding oomycete growth (Derevnina et al. 2016). *P. infestans*encodes six conserved elicitin proteins (Jiang et al. 2006; Cooke et al. 2012). Through map-based cloning, a receptor-like protein (RLP) ELICITIN RESPONSE (ELR) from wild potatoes was isolated; the extracellular domain of ELR recognizes the conserved domain of elicitins (Du et al. 2015). Because RLPs lack a cytoplasmic signaling domain, they form heterodimers with SUPPRESSOR OF BIR1-1 (SOBIR1) to participate in the immune responses (Gust and Felix 2014). The kinase activity of SOBIR1 is necessary for plant immunity triggered by elicitins, such as INF1 (Domazakis et al. 2018). Upon INF1 stimulation, ELR forms heterodimers with SOBIR1 and then recruits BAK1, thereby activating downstream signaling (Du et al. 2015; Domazakis et al. 2018). Similar to the PTI pattern in *Arabidopsis*, elicitins induce potato ELR to detect elicitins and form trimers with SOBIR1 and BAK1, eliciting a PTI immune response to resist the invasion of *P. infestans*.

Other receptor-like kinases (RLKs) have been identified to enhance the resistance of potatoes to *P. infestans*. Research on *STRUBBELIG-RECEPTOR FAMILY* (*SRF*) homologous genes in potatoes indicated that *StLRPK1* is significantly induced following infection by *P. infestans *(Wu et al. 2009). Overexpression of*StLRPK1* enhances resistance to *P. infestans*, with the interaction between StLPRK1 and BAK1 playing a crucial role in the potato’s defensive response against *P. infestans *(Wang et al. 2018). In *Arabidopsis*, L-type lectin receptor kinase-I0.9 (LecRK-I0.9) kinase can recognize and combat *Phytophthora *infections (Senchou et al. 2004; Bouwmeester et al. 2011). The extracellular lectin domain of LecRK-I0.9 binds to the RGD tripeptide motif of the IPI-O effector released by*P. infestans *to transmit signals and initiate downstream immune responses (Gouget et al. 2006). The *Arabidopsis lecrk-I.9* mutant exhibits strong sensitivity to *P. infestans*, and overexpressing *AtLecRK-I0.9 *enhances the plant’s resistance to oomycetes (Bouwmeester et al. 2011). Further overexpressing *Arabidopsis LecRK-I.9* in potato substantially increased the resistance to *P. infestans *(Bouwmeester et al. 2014). The *StLecRK-IV 0.1* gene was considerably significantly downregulated in *P. infestans* infection, and silencing *StLecRK-IV.1* in potato reduced the infection plaque of *P. infestans *(Guo et al. 2022). Molecular studies have identified an interaction between StLecRK-IV.1 and StTET8, and coexpression of *StLecRK-IV.1* and *StTET8 *can disrupt the stability of StTET8 and reduce resistance to late blight (Guo et al. 2022). Recently, Pep-13 receptor unit (PERU) was isolated from wild potato varieties, which can recognize small peptides Pep13 secreted by*P. infestans*. Binding of PERU to Pep13 facilitates the formation of a PERU-BAK1 complex, triggering an immune response and enhancing immunity to *P. infestans* (Torres Ascurra et al. 2023).

RLKs help transmit signals from the outside to the inside of plant cells, activating plant PTI responses. These processes involve increased ROS production and MAPK kinase activation. Potatoes’ MAPKs play a role in resisting infection of *P. infestans *(Majeed et al. 2022). An MAPK cascade involving StMEK1-StMPK1/StWIPK has been identified in response to *P. infestans *(Yamamizo et al. 2006). Overexpressing *StMEK1* stimulated StRBOHC and StRBOHD peroxidase activities, increased ROS levels, resulting in enhanced resistance to *P. infestans *(Yamamizo et al. 2006). StMPK7 is phosphorylated by StMKK1 to regulate the immune response to *P. infestans *(Zhang et al. 2021b). The StLRPK1-BAK1 complex can transmit downstream signals to MEK2 and WIPK, components of the MAPKs cascade, to activate PTI immunity (Wang et al. 2018).

When pathogens infect potatoes, they secrete effectors into plant cells to suppress immune responses and enhance the success rate of infection (Jones and Dangl 2006). The effectors from *P. infestans *mainly fall into two categories: crinkler (CRN) and RxLR (Kamoun et al. 2006). The genome of *P. infestans *encodes over 500 RxLR effectors, with diversity in sequence and expression across strains (Haas et al. 2009; Anderson et al. 2015). The study of effectors is widely considered key to understand the disease-transmitting mechanism of oomycetes. Effectors specifically target various proteins in host plant cells, thereby altering the stability, activity, or subcellular localization of target proteins and interfering with the normal immune response of plants (He et al. 2020). Potatoes have evolved resistance (R) genes to counter effectors, triggering strong ETI immune processes. These effectors, recognized by *R *genes, are named avirulence proteins (AVRs, Vleeshouwers et al. 2011). The main proteins like AVR1, AVR2, AVR3a, AVR4, AVR8, AVRblb1, AVRblb2, PexRD12/31, have been identified and their functions are continually being studied (Vleeshouwers et al. 2011; Monino-Lopez et al. 2021).

AVR1 interacts with Sec5, an exocyst component, stabilizing it and disrupting the secretion of pathogenesis-related protein-1 (PR1) and callose deposition (Du et al. 2015b). Stable Sec5 interferes with vesicular transport to inhibit normal immune response (Du et al. 2015b). AVR2 interacts with BSL1 (BSU-LIKE PROTEIN1), which is required for R2 protein’s recognition of AVR2 and resistance (Saunders et al. 2012). AVR2 serves as a crosstalk between BR and immune signals. The bHLH transcription factor StCHL1 is induced under both BR and AVR2 conditions (Turnbull et al. 2017) and simultaneous expression of *StCHL1* and *AVR2*inhibits plant INF1-indcued immunity (Turnbull et al. 2017). AVR3a is the first cloned and isolated effector of *P. infestans *(Armstrong et al. 2005), which inhibits INF1-mediated cell hypersensitivity by binding to and stabilizing an E3 ubiquitin ligase CMPG1 (Bos et al. 2010). In addition, AVR3a interacts with GTPase Dynamin-Related Protein 2, which mediates receptor protein endocytosis, thereby inhibiting ROS bursts triggered by PTI immunity (Chaparro-Garcia et al. 2015). The different targets of AVR3a indicate that AVR3a can inhibit PTI signals in various ways. AVR8 binds to and degrades potato desumoylating isopeptidase 2 (StDeSI2), through 26S proteasomes, thereby attenuating PTI responses (Jiang et al. 2023). Continuous research on the identification of effectors and their mechanisms can significantly broaden the knowledge on the plant-pathogen interaction network. For instance, PexRD54 effector binds to the autophagy-associated protein ATG8CL (Dagdas et al. 2016), mimicking the starvation-induced autophagy to break the balance of endomembrane trafficking at the pathogen interface (Pandey et al. 2021). Pi02860 effector binds to the E3 ubiquitin ligase NPH3/RPT2-LIKE1 protein (NRL1) to enhance the interaction between NRL1 and a guanine nucleotide exchange factor called SWAP70, promoting SWAP70 degradation and suppressing immune responses (He et al. 2018). Pi04314 effector interacts with protein phosphatase 1 catalytic (PP1c) isoforms, attenuating immunity responses by changing their localization (Boevink et al. 2016). PiSFI3/Pi06087/PexRD16 effector interacts with a U-box-kinase protein (StUBK) to suppress the UBK-related PTI immunity response (He et al. 2019). Pi22926 and PexRD2 target MAPKKK kinase to inhibit the kinase activity, thereby subverting plant immunity (King et al. 2014; Ren et al.  2019).

The integration of genomics, proteomics, and microscopy has substantially advanced our understanding of effectors’ biological functions. Using core RXLR effector factors, protein mass spectrometry identified proteins interacting with effectors, constructing a effector-host protein interaction network (Petre et al. 2021). Extensive interactions between RXLR effectors and vesicular transport system components highlight the crucial role of vesicular transport in the relationship between Phytophthora and plant cells (Petre et al. 2021). Previous studies on effector entry into cells suggested that the RXLR motif's high sequence conservation with plant proteins might allow effectors to perform similar functions and evade the plant immune system (Birch et al. 2008). Inhibiting endocytosis-related proteins reduced Phytophthora infection efficacy and effector translocation into cells (Wang et al. 2023a, b). These results demonstrate that RXLR effectors enter cells through clathrin-mediated endocytosis.

The findings of immune studies in *Arabidopsis *indicate that immune responses in potatoes also conform to the “zig-zag” model (Jones and Dangl 2006). The identification of other homologous pattern recognition receptors, ELR and PERU, in potatoes establishes a solid foundation for delineating PTI immune pathways and exploring additional signaling components to enrich the pathway (Du et al. 2015a; Torres Ascurra et al. 2023). Current research in ETI immunity mainly focuses on elucidating the mechanisms of RxLR effectors actions and their target proteins (Anderson et al. 2015), while an increasing number of molecular mechanisms underlying effector-target interactions are being uncovered. Research methodologies are advancing toward greater efficiency, diversity, and comprehensiveness, transitioning from analyzing single effector-target interactions to exploring multi-target interactions and conducting omics analyses of multiple effectors, which is expected to reveal novel effector mechanisms, enriching our understanding of molecular mechanisms in potato disease resistance and offering valuable insights into disease-resistant potato breeding.

#### Potato virus disease, PVY

Viruses represent a substantial threat to the normal growth, harvesting, and sowing of potatoes. Over time, plants intricate monitoring mechanisms to identify and counteract viral invaders. They use diverse approaches, including RNA silencing, transcriptional inhibition, and *R *gene-mediated defense, to resist viruses (Soosaar et al. 2005). Globally, potatoes are susceptible to over fifty viral species (Kreuze et al. 2020), with six major threats: *Potato virus Y* (PVY), *Potato virus X* (PVX), *Potato virus S* (PVS), *Potato virus A* (PVA), *Potato leafroll virus* (PLRV), and *Potato virus M*(PVM) (Wang et al. 2011), among which PVY have caused severe economic losses in the potato industry over the past three decades, making it a focal point of research (Kreuze et al. 2020).

Exploring resistance genes is a key strategy in combating PVY. The *R* and *N* genes are two types of PVY resistance genes in potatoes (Valkonen et al. 1996). The *N *gene triggers a hypersensitive resistance response (HR) in potatoes and exhibits strain specificity (Singh et al. 2008). Specific *N* genes, such as *Ny*, *Nc*, and *Nz*, confer resistance to different PVY strains: PVY^O^, PVY^C^, and PVY^Z^, respectively (Singh et al. 2008). HR results in localized leaf necrosis, preventing virus from spreading to other plant tissues, although it does not completely stop virus replication. Conversely, the extreme resistance (ER) response triggered by the *R *gene offers broad-spectrum, strong, and long-lasting resistance (Baebler et al. 2020). ER inhibits virus proliferation in the early stages of virus infection, resulting in minimal signs of infection in leaves (Ross et al. 2021). Currently, 10 PVY disease resistance genes have been identified in potatoes, with few members cloned and studied. The Ry_*sto*_ gene encodes a TIR-NLR immune receptor and stable expression of Ry_*sto*_ protein in two *Solanaceae *plants, potato and tobacco, effectively limited the transmission of PVY and PVA viruses (Grech-Baran et al. 2020). Ry_*sto*_ directly binds to the coat protein (CP) of PVY virus, exhibiting ER resistance. Given the conservation of virus CP proteins, Ry_*sto*_ is speculated to recognize at least 10 significant viruses (Grech-Baran et al. 2022).

The eIF4E initiation factor is a crucial resistance protein in potatoes. eIF4E binds to viruses and exerts its effects through transcription pathways, and most clones of recessive resistance genes are variants of eIF4E or its isoforms (Truniger and Aranda 2009; Wang and Krishnaswamy 2012). Potato viruses use eIF4E to synthesize their proteins; however, isoforms of eIF4E can evade viral manipulation, contributing to resistance (Sanfaçon 2015). Numerous eIF4E variants exist in the natural population and overexpression of *eIF4E *can substantially enhance the potatoes resistance to viruses without adversely affecting normal crop growth (Gutierrez Sanchez et al. 2020).

In addition to using resistance genes, potatoes employ a strategy of restricting virus spread as a key defense mechanism against viral infection. This is an active area of research in plant-virus interaction. Plasmodesmata (PD) serve as vital channels between adjacent plant cells, enabling short-distance signaling transmission and material exchange (Lucas and Lee 2004). They also facilitate the movement of viruses (Liu et al. 2021). REMORIN (REM) proteins, associated with plant plasma membrane (PM), are integral to the PD-PM subcompartment and involved in virus cell-to-cell movement (Raffaele et al. 2009). Overexpression of *StREM1.3 *limited the dilation effect of triple gene block proteins1 (TGBp1) on PD (Perraki et al.  2014). When infected with PVX, the viral coat protein and TGBp1 interact with REM1.3, leading to its phosphorylation. This phosphorylation triggers callose deposition at PD, reducing their permeability and thus hindering viral spread (Perraki et al. 2018).

The detrimental effects of potato viruses on crop health and yield are substantial, making the identification of resistance genes a crucial aspect. Although numerous PVY resistance genes have been mapped, only few have been cloned and applied. As sequencing technologies and microscopic techniques continue to evolve, the identification and characterization of virus resistance genes and their underlying mechanisms are expected to accelerate, which will enhance our understanding of potato virus diseases and support the development of new potato varieties with effective resistance to viral pathogens, offering more suitable solutions for combating these agricultural challenges.

#### Abiotic stress

**Fig. 7:**
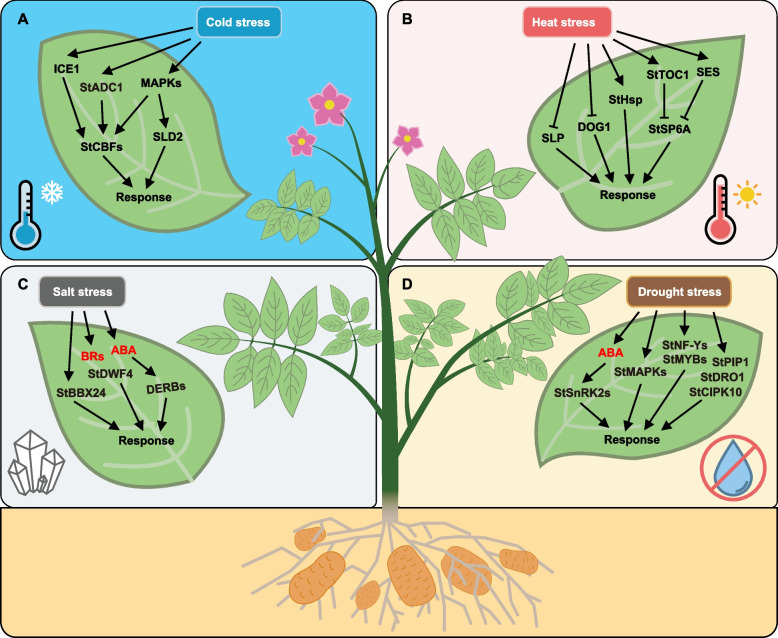
Regulatory network of cold (**A**), heat (**B**), salt (**C**) and drought (**D**) stresses in potatoes. **A** The upregulated ICE1 and ADC1 promote transcriptional activity of StCBFs under cold stress. MAPKs kinase activities are activated to promote *StCBFs* and *SLD2* expression. **B** Under heat stress, induced *SES* and *StTOC1* downregulate *StSP6A*. Heat suppresses the transcriptions of *DOG1* and *SLP* while promotes that of *StHsps*. **C** Under salt stress, ABA plays a crucial role by upregulating *DREBs*. BRs, *StDWF4* and *StBBX24* promotes salt stress-related genes and enhances resistance to salt. **D** Under drought, ABA and MAPKs are induced and ABA activates SnRKs kinase activity. Transcription factors (e.g., NF-Ys and MYBs), StPIP1, StDRO1, and StCIPKs are also involved in drought response

Abiotic stresses including high temperature, drought, mineral deficiencies or toxicities, salinity, and low temperature, significantly affect the normal growth of potatoes (Fig. 7, Demirel 2023). Currently, related research mainly focuses on gene function identification, highlighting the necessity for a deeper understanding of the specific molecular mechanisms of related genes.

#### Low temperature

Potatoes thrive in cool environments but are vulnerable to frost and freezing, which detrimentally affect their yield and quality (Simon 1993). Low temperatures primarily damage cell membranes and hinder photosynthesis and other physiological processes (Guan et al. 2023). Transcriptome sequencing of the diploid wild species *S. commersonii* under low temperature conditions revealed the upregulation of numerous genes, including known cold stress-related genes such as *INDUCER OF CBF EXPRESSION 1* (*ICE1*) and *CBF3 *(Aversano et al. 2015). In addition, analysis combining transcriptomics and metabolomics demonstrated an increased expression of *StADC1 *under low temperature stress, leading to elevated putrescine levels (Kou et al. 2018). Both the exogenous application of putrescine and overexpression of *StADC1* activated *StCBF *transcription activity, thereby enhancing the cold resistance of potatoes (Kou et al. 2018). Overexpression of *StCBF3* in *Arabidopsis *considerably improved its tolerance to low temperature (Li et al. 2018). In cold-tolerant potato species*S. acaule*, the expression of *SaMKK2* was particularly increased, and SaMKK2 interacted with SaMAPK4/7, promoting the expression of *CBFs* and *SLD2 *and thus enhancing the resistance of potatoes to cold stress (Chen et al. 2022).

#### High temperature

As a cool-loving crop, potatoes are susceptible to heat stress, which substantially affects their growth, development, and tuber formation process, eventually leading to decreased tuber yield and quality (Lafta and Lorenzen 1995). Previous studies have examined the role of StSP6A in the photoperiod regulation of tuber formation, while transcription data analyses have revealed a substantial decrease in *StSP6A *expression under high temperature conditions (Hancock et al. 2014; Morris et al. 2019; Lehretz et al. 2019), revealing its unexpected function under heat stress. Research showed that the core clock protein TIMING OF CAB EXPRESSION1 (StTOC1) acts as a temperature-responsive transcription factor, inhibiting *StSP6A *expression under high temperature conditions (Hancock 2014; Morris et al. 2019). Silenced expression of *StTOC1* caused an increased transcription of *StSP6A*, thereby increasing tuber yield under high temperature conditions (Morris et al. 2019). Furthermore, high temperature conditions lead to the induction of *SES*, which reduces the expression of *StSP6A *(Park et al. 2022). By contrast, interference with SES can increase the tuber yield of potatoes under high temperatures (Park et al. 2022).

Transcriptome analysis revealed the presence of numerous heat stress-related genes in potatoes. A study examining the response of 18 potato varieties to heat stress found that the dormancy-related genes *DELAY OF GENEATION 1* (*DOG1*) and *SUBTILISIN-LIKE PROTEASE* (*SLP*) were significantly downregulated (Zhang et al. 2021a). Heat stress treatment led to the enrichment of the GA biosynthesis pathway, suggesting the involvement of GA in heat sprouting and dormancy changes in tubers (Zhang et al. 2021a). Moreover, transcriptome analysis in potato leaves demonstrated an increase in the expression of the heat shock proteins StHsp26-CP and StHsp70 (Tang et al. 2020).

#### Salinity

Because of excessive fertilizer and pesticide usage in agriculture, and intensive land utilization, soil salinization is becoming increasingly prevalent, especially in arid regions where reduced precipitation and soil moisture evaporation exacerbate the issue. Salt stress inhibits the growth of potato, leading to decreased yields. However, potatoes have developed various ways to cope with salinity (Chourasia et al. 2021).

ABA plays a crucial role in potato resistance to salt stress. Under salt stress, dehydration responsive element binding protein (StDREB2) is rapidly induced through the ABA signaling pathway, and StDERB2 overexpression led to increased tolerance to salt stress (Bouaziz et al. 2012). Similarly, potatoes with heterologous expression of *AtDREB1A *showed an increase in salt resistance (Behnam et al. 2006). Applying BRs during potato growth can increase the proportion of K ^+^ /Na ^+^ ions in the tissue and improve salt tolerance (Hu et al. 2016). Overexpression of *StDWF4 *enhanced BR biosynthesis and thus salt tolerance in potatoes (Zhou et al. 2018). Heterologous expression of *Arabidopsis high-affinity potassium transporter 1* (*AtHKT1*) in potatoes increased the intercellular K ^+^ /Na ^+^ ratio, improved water utilization efficiency, and reduced the transpiration rate, effectively increasing salt tolerance (Wang et al. 2019). BBX, a type of B-box zinc finger protein, also known as CO is a well-studied protein controlling flowering (Abelenda et al. 2016). Suppressed expression of *StBBX24 *led to greater sensitivity to salt stress, whereas its overexpression resulted in a salt-tolerant phenotype (Kiełbowicz-Matuk et al. 2022).

#### Drought

Potatoes can provide more nutrients than other major crops under the same water-use conditions (Renault and Wallender 2000). However, their root structure leads susceptibility to drought stress (Monneveux et al. 2013). ABA plays a crucial role in potato drought resistance, which regulates stomatal movement, affects the distribution of dry matter and potato yield (Liu et al. 2005). Moderate drought condition increases ABA levels in potato roots, leaves, and stolons, promoting tuber formation and increasing potato weight (Xu et al. 1998). Sucrose nonfermenting 1-related protein kinases 2 (SnRK2) subfamily genes are mainly activated in response to abiotic stress. In the presence of ABA, activated SnRK2s phosphorylate downstream transcription factors, regulating the expression of downstream response genes and thus improving drought resistance (Chen et al. 2020). The expression level of *StSnRK2 *family genes was substantially upregulated under drought stress, and the stable transformation of StSnRK2 in tobacco resulted in stronger drought resistance (Yao et al. 2023).

MAPKs also play a significant role in drought stress, beyond their function in biotic stress. Drought conditions substantially upregulated the expression of *StMAPK11*, enhancing the antioxidant activity of potatoes and drought resistance through photosynthesis (Zhang et al. 2021a, b). Moreover, a study identified 22 drought-responsive genes across different potato species, with MAPKKK15 showing substantial upregulation at the transcriptional level under water deficit condition (Pieczynski et al. 2018).

Various transcriptional factors play roles in response to drought stress. Nuclear factor Y (NF-Y) is a common family of transcription factors in eukaryotes and StNF-A7 enhances drought tolerance by reducing leaf water loss (Na et al. 2017). Overexpression of *StNF-YB3.1 *promoted ABA-mediated stomatal closure, leading to a decrease in tuber yield (Xuanyuan et al. 2017). Overexpressing*StNF-YC9*increased potato tolerance to water deficiency by enhancing photosynthesis and antioxidant enzyme activity (Li et al. 2021). The MYB gene family, comprising 233 genes in potatoes, is predominantly activated under drought stress (Li et al. 2019). *StMYB1R-1* was identified as a stress-response gene and overexpression of *StMYB1R-1 *improved drought tolerance and enhanced drought-regulated gene expression (Shin et al. 2011). Overexpressing *StMYB33 *improved potato drought resistance through regulating ABA signaling pathway (Wyrzykowska et al. 2022). Overexpression of *AtDREB1A *improved drought resistance, marked by increased activities of superoxide dismutase, catalase, and peroxidase (Jia et al. 2016).

Overexpression of *AtYUCCA6 *reduced water loss, enhancing drought resistance and reducing ROS levels in potato leaves (Kim et al. 2013). The *DEEPER ROOTING 1* (*DRO1*) regulates grain yield and root system under drought condition in rice (Uga et al. 2013) and the potato homologous gene *StDRO1* affects the root structure and restores the tolerance of *Atdro1*to drought (Sun et al. 2022a, b). Similarly, overexpressing *StPIP1*, a potato water channel plasma membrane intrinsic protein, improved drought resistance by maintaining photosynthesis, stomatal conductance, and water use efficiency (Wang et al. 2017). Under ABA and drought conditions, the expression of *StCIPK10 *significantly increased, which contributes to the enhancement of ROS scavenging and accumulation of osmolytes, aiding in the adaptation to drought stress of potatoes (Ma et al. 2021a, b).

Compared with *Arabidopsis*, studies on abiotic stress in potatoes have not yet established a relatively complete regulatory network. This gap is partly determined by the inherent characteristics of potatoes. The tolerance to environmental factors and related impact are both worth to be analyzed with the widespread application of multi-dimensional omics, a comprehensive understanding of relationship among various environmental factors is expected, which can be efficiently applied in potato molecular breeding to address global environmental threats.

### Application of biotechnology in potato trait improvement

Due to the significant edible and economic value of potatoes, enhancing their traits has always been a focal point for researchers. However, the complexity of breeding is heightened by the tetraploid nature, high genetic heterozygosity, limited genetic diversity, and intricate genetic architecture of key traits in most cultivated potatoes, making traditional breeding laborious and inefficient. Genetic engineering offers a remedy by enabling the integration of one or more foreign genes into the plant genome, thereby inducing targeted genetic modifications without altering the overall genetic makeup. This approach addresses the limitations of conventional breeding, like extended cycles and inefficiency, presenting a novel pathway for developing new varieties. Since the initial introduction of a transgenic potato plant via *Agrobacterium tumefaciens*in the 1980s (Ooms et al. 1986), this technology has significantly advanced potato breeding. It facilitates the incorporation of genes that bolster resistance to pathogens and pests, enrich nutritional profiles by boosting levels of proteins, vitamins, carotenoids, and lipids, and minimize harmful substances like acrylamide and glycoalkaloids. Moreover, genetically modified potatoes have gained attention for their potential to produce modified starches, lipids, and recombinant proteins, including pharmaceuticals and vaccines (Hameed et al. 2018). A recent innovation by Mei et al. introduced an effective planta transformation technique leveraging plants' innate regenerative capabilities (Mei et al. 2024). By injecting *A. tumefaciens *directly into plant meristems, transfected tissues are induced, with subsequent vegetative propagation yielding stable transgenic plants, which bypasses the need for tissue culture, offering substantially higher transformation efficiencies than conventional methods and has been successfully applied to potatoes. Currently, several genetically modified potato varieties have been commercialized, including the NewLeaf™ and NewLeaf Plus™ varieties developed by Monsanto® in the late 1990s for resistance to the Colorado potato beetle and potato leafroll virus (PLRV) (Lawson et al. 2001). Another example is the Innate® potato, developed by J. R. Simplot Company, which features reduced enzymatic browning and lower acrylamide levels when processed (Richael 2021). Most GM potato varieties have been produced using transgenic and RNA interference (RNAi) techniques (Hameed et al. 2018; Richael 2021).

In recent years, genome editing technologies have emerged as novel biotechnological approaches for crop breeding, garnering attention for their precision and efficiency in generating targeted genetic modifications to achieve desired traits (Baltes et al. 2017; Chen et al. 2019; Zhu et al. 2020a). Utilizing site-specific nucleases (SSNs) such as Zinc Finger Nucleases (ZFNs), Transcription Activator-Like Effector Nucleases (TALENs), and the Clustered Regularly Interspaced Short Palindromic Repeats (CRISPR) along with CRISPR-associated proteins (CRISPR/Cas), genome editing introduces precise genetic modifications. The detailed potato genome sequence and advanced transformation systems position the potato as an ideal subject for genome editing to enhance traits essential for more sustainable production (Hameed et al. 2018; Nadakuduti 2018). Since the initial demonstration of CRISPR/Cas9-mediated genome editing in potato in 2015 (Wang et al. 2015a), this technique has been leveraged in numerous studies to enhance traits of potatoes, including improving nutritional quality, modifying tuber starch composition, enhancing post-harvest quality, increasing biotic stress tolerance, and addressing reproductive self-incompatibility issues (Table 1).

**Table 1:** Applications of genome editing for agronomic traits improvement in potato. T, transcription activator-like effector nucleases (TALENs); C9, clustered regularly interspaced short palindromic repeats/CRISPR associated 9 (CRISPR/Cas9); C13, CRISPR/Cas13; RNP, protoplast transfection with ribonucleoproteins; CBE, cytosine base editor; PE, Prime Editor

Edit tool	Delivery approach	Genotype	Target gene	Objective	References
T	*A. tumefaciens*	cv. Sassy	Sterol side chain reductase 2 (SSR2)	Reduction of SGA	Sawai et al. 2014
T	Protoplast	cv. Desiree	Acetolactate synthase (ALS)	Herbicide resistance	Nicolia et al. 2015
T	Protoplast	cv. Ranger Russet	Vacuolar invertase (VInv)	Reduction of cold-induced sweetening (CIS)	Clasen et al. 2016
T	*A. tumefaciens*	cv. Ranger Russet	Acetolactate synthase (StALS)	Herbicide resistance	Forsyth et al. 2016
T	*A. tumefaciens*	cv. Sayaka	Granule-bound starch synthase (GBSS)	Improving starch quality	Kusano et al. 2016
T	*A. tumefaciens*	cv. Shepody and cv. Russet Burbank	GBSS and vacuolar invertase (VInv)	Improving starch quality	Ma et al. 2017
T & C9	*A. tumefaciens*	cv. Desiree and diploid self-incompatible breeding line, MSX914-10	ALS	Herbicide resistance	Butler et al. 2016
C9	*A. tumefaciens*	DM	Phytoene desaturase (PDS) and StAA2 gene (an Aux/IAA gene)	Proof of concept	Wang et al. 2015a, b
C9	*A. tumefaciens*	cv. Desiree and diploid self-incompatible breeding line, MSX914-10	ALS	Proof of concept	Butler et al. 2015
C9	*A. tumefaciens*	cv. Desiree	Transcription factor gene MYB44	Functional genomics	Zhou et al. 2017
C9	*Protoplast*	cv. Kuras	GBSS	Modification of starch composition. High amylopectin	Andersson et al. 2017
C9	*A. tumefaciens*	S. tuberosum group Phureja S15-65 clone	S-locus RNase (S-RNase)	Elimination of reproductive self-incompatibility	Ye et al. 2018
C9	*A. tumefaciens*	DRH-195 and DRH-310	S-RNase	Elimination of reproductive self-incompatibility	Enciso-Rodriguez et al. 2019
C9	*A. rhizogenes*	cv. Mayqueen	Steroid 16a-hydroxylase (St16DOX)	Nutritional quality. Reduction of toxic steroidal glycoalkaloids (SGAs)	Nakayasu et al. 2018
C9	*RNP*	cv. Kuras	GBSS	Modification of starch composition. High amylopectin	Andersson et al. 2018
C9	*A. tumefaciens*	cv. Sayaka	GBSS	Optimization of Cas9 expression with d-Mac3 translational enhancer	Kusano et al. 2018
C9	*Protoplast and A. tumefaciens*	cv. Desiree	Starch branching enzymes (SBE1 and SBE2)	Modification of starch composition /nutritional quality. High amylose and longer amylopectin chains	Tuncel et al. 2019
C9	*Protoplast*	cv. Desiree and cv. Wotan	GBSS	Modification of starch composition. High amylopectin	Johansen et al. 2019
C9	*RNP*	cv. Desiree	Polyphenol oxidase 2 (StPPO2)	Post-harvest quality. Reduction of enzymatic browning	González et al. 2020
C9	*A. tumefaciens and RNP*	cv. Desiree	StPPO2	Reduction of enzymatic browning	González et al. 2021
C9	*A. tumefaciens*	cv. Desiree and cv. King Edward	S-genes (StMLO1, StHDS,StTTM2, StDND1, StCHL1, StDMR6-1 and StDMR6-2)	Biotic stress tolerance. Resistance to *Phytophthora infestans*	Kieu et al. 2021
C9	*RNP*	cv. Desiree	SBE1 and SBE2	Modification of starch composition /nutritional quality. High amylose and longer amylopectin chains	Zhao et al. 2021
C9	*A. tumefaciens*	cv. Yukon Gold TXYG79	GBSSI	Decrease in amylose content	Toinga-Villafuerte et al. 2022
C9	*A. tumefaciens*	cv. Sayaka	SBE3	Low amylose	Takeuchi et al. 2021
C9	*A. tumefaciens*	cv. Desiree	SS6	N/A	Sevestre et al. 2020
C9	*Protoplast*	cv. Wotan, Saturna	GWD1	N/A	Carlsen et al. 2022
C9	*A. tumefaciens*	cv. Desiree	PDS	Bleached (albino) leaves	Bánfalvi et al. 2020
C9	*A. tumefaciens*	cv. DMF1	PDS	Bleached whole plantlets	Butler et al. 2020
C9	*A. tumefaciens*	cv. Atlantic	SSR2	66% of WT tuber	Zheng et al. 2021
C9	*Protoplast*	cv. Desiree	eIF4E1	Partial resistance to PVY	Lucioli et al. 2022
C9	*A. tumefaciens*	cv. B665 and B663	Sli	Self-incompatible plants	Eggers et al. 2021
C9	*A. tumefaciens*	cv. Phureja S15–65	IT1	Loss of tuberization	Tang et al. 2022
C9	*A. tumefaciens*	cv. Russet Burbank	CCOAOMT	Improved resistance to late blight	Hegde et al. 2021
C13	*A. tumefaciens*	cv. Desiree	P3, CI, NIB, CP	Improved resistance to PVY	Zhan et al. 2019
CBE	*A. tumefaciens*	cv. Desiree	ALS	Herbicide resistance	Veillet et al. 2019b
CBE	*A. tumefaciens*	cv. Desiree	GBSS and ALS	Construction of A3A-PBE cytosine base editor	Zong et al. 2018
C & CBE	*Protoplast & A. tumefaciens*	cv. Desiree and cv. Furia	GBSS	Modification of starch composition. High amylopectin	Veillet et al. 2019a
C & CBE	*A. tumefaciens*	cv. Desiree	GBSS and Downy Mildew Resistant 6 (StDMR6-1)	Proof of concept	Veillet et al. 2020
PE2	*A. tumefaciens*	cv. Desiree	ALS1	Improved herbicide resistance	Perroud et al. 2022

### Improving starch quality

Potato consists of amylose (about 20–30%) and amylopectin (about 70–80%) and is a significant source of starch for both food and industrial applications (Fajardo et al. 2013). The amylose/amylopectin ratio in tuber starch varies among cultivars but is typically around 1:4. This ratio is crucial as it influences the physical and chemical properties of starch, affecting its suitability for various dietary and industrial uses (Zeeman et al. 2010). Recent advancements have utilized CRISPR/Cas system-mediated genome editing to successfully modify starch composition in potatoes for diverse applications.

Amylose-free starch, particularly amylopectin, is used as an adhesive in the paper industry and as a thickening, bulking, or coating agent in the food industry. To produce potatoes with amylose-free starch or a high amylopectin/amylose ratio, the *GBSS1 *gene responsible for amylose synthesis can be targeted. This gene has been disrupted using various methods, including antisense technology, RNAi, TALENs, and CRISPR/Cas9 (Visser et al. 1991; Otani et al. 2007; Andersson et al. 2017; Kusano et al. 2018). To improve starch quality, (Andersson et al. 2017) targeted all the alleles of *GBSS* genes in tetraploid potato through the transient expression of CRISPR/Cas9 system in protoplasts. Three mutated lines were identified to harbor inserts of original plasmid. A regenerant identified from a specific transformation event contained all copies of the mutated *GBSS1 *gene. Moreover, starch from microtubers of this line exhibited the waxy phenotype (Andersson et al. 2017). Subsequently, CRISPR/Cas9 ribonucleoprotein (RNP) was used to target the *GBSS *gene for DNA-free genome editing. RNPs were produced synthetically (cr-RNP) and in vitro (ivt-RNP), with mutation frequencies of 9% and 25%, respectively. All the mutated lines produced from cr-RNP were transgene-free, and mutations were induced in all four alleles, resulting in a complete knockout of the GBSS enzyme function (Andersson et al. 2018). Both studies have demonstrated that GBSS1 can be completely mutagenized through the delivery of RNPs or plasmid DNA into protoplasts and that transgene free waxy tubers can be obtained through the subsequent regeneration of potato plants. The efficiency of CRISPR/Cas9 to target the *GBSS* gene was enhanced using translational enhancer *dMac3*, and amylose-reduced lines were obtained using the CRISPR/Cas system for the stable transformation of Sayaka (Kusano et al. 2018).

In another study, substituting the AtU6 promoter with the endogenous potato U6 promoter to drive the expression of sgRNAs improved the efficiency of CRISPR/Cas9-mediated *GBSS*gene editing (Johansen et al. 2019). Toinga-Villafuerte et al. (2022) produced a stable knockout line of *GBSS1* through the *Agrobacterium*-mediated transformation of a yellow potato cultivar Yukon Gold, which essentially had no detectable amylose in tubers. Additionally, the role of base editors causing specific and efficient nucleotide substitution can widen the scope of gene editing. It is demonstrated that DNA substitutions in the locus encoding the catalytic domain KTGGL of the potato GBSS, which was generated by cytidine base editor (CBE), were sufficient to produce a loss-of-function allele (Veillet et al. 2019b). Two mutants, out of 48 stable regenerants, had an unusual C-17 to G-17 conversion in all alleles and had no other unintended mutations at the target site. This nucleotide substitution resulted in the L99V mutation in the KTGGL motif and the mutant microtuber lacked amylose.

Amylose-rich and digestion-resistant starch can be consumed as a healthier alternative to reduce calorie intake, improve insulin resistance, and promote gut health (Keenan et al. 2015). To increase the content of resistant starch in potatoes, studies have focused on reducing the activity of starch branching enzymes 1 and 2 (SBE1 and SBE2), which are crucial for inserting α-1,6 glycosidic bonds in amylopectin. RNAi and antisense RNA approaches substantially reduced SBE activity in tubers, resulting in starches that were high in amylose content and resistant to digestion (Schwall et al. 2000; Andersson et al. 2006). Recent studies have used CRISPR/Cas9-mediated mutagenesis to target *SBE *genes, producing transgene-free tubers rich in amylose and resistant starch. Tuncel et al. (2019) targeted *SBE1*, *SBE2*, or both genes through conventional *Agrobacterium*-mediated transformation or PEG-mediated transient protoplast transfection to deliver CRISPR/Cas9 components. Transgene-free potatoes with different levels of long amylopectin chains and/or amylose were created. Mutants with a decrease in the SBE2 protein level alone exhibited increased normal amylopectin chain length and starch granule initiation, whereas those decreases in both SBEs displayed an extreme phenotype with reduced amylopectin branching during granule development. Another study using CRISPR/Cas RNP-method revealed that starches of lines with mutations in all the alleles editing SBE1 and SBE2 were composed of amylose (> 95%) and had no detectable amylopectin (Zhao et al. 2021). Both studies have indicated that the transient expression of sgRNA/Cas9 constructs and the delivery of RNP complexes to protoplasts are promising tools for producing transgene-free plants with mutations in*SBE* genes.

### Reducing cold-induced sweetening

To reduce sprouting and extend post-harvest shelf life, harvested potato tubers are commonly stored at low temperatures (approximately 4 °C). However, during such storage, they often accumulate reducing sugars, such as glucose and fructose, due to starch conversion, a phenomenon known as cold-induced sweetening (CIS) (Dale and Bradshaw 2003), which posing a significant challenge for the subsequent processing of potatoes. When subjected to high-temperature frying, the reducing sugars undergo a non-enzymatic Maillard reaction with free amino acids, causing the fried slices to darken and develop a bitter taste, which are not consumer friendly (Mottram et al. 2002; Pedreschi et al. 2005). Moreover, the formation of a carcinogenic compound acrylamide in this reaction poses serious health risks (Bethke and Bussan 2013; Sowokinos et al. 2004; Tareke et al. 2002; Pedreschi et al. 2005).

Vacuolar invertase (VInv) is one of the enzymes involved in cold-induced sweetening, responsible for converting sucrose to glucose and fructose (Sowokinos et al. 2004; Bhaskar et al. 2010; Zhang et al. 2017). Transcription of *VInv *is significantly upregulated under cold conditions, while downregulated at normal temperatures (Zrenner et al. 1996; Bhaskar et al. 2010). Decreasing reducing sugars is the effective way to suppress negative effect of CIS (Becalski 2004; Zhu et al. 2016), and previous studies have revealed overexpressing an invertase inhibitor from tobacco in potatoes (Greiner et al. 1999), or using antisense RNA (asRNA), RNAi or CRISPR-Cas9 to suppress the *VInv *transcription or reduce its activity and cold-induced reducing sugars accumulation, could effectively reduce CIS (Zrenner et al. 1996; Bhaskar et al. 2010; Wu et al. 2011; Clasen et al. 2016; Ly et al. 2023). Recently, an intronic enhancer VInvIn2En, governing cold-induced expression of *VInv* was dissected in potato to upregulate *StVInv1 *under cold conditions (Zhu et al. 2024). Remarkably, 5 out of 18 transformed lines exhibited nearly complete silencing of the *VInv *gene, with minimal or undetectable CIS activity (Hou et al. 2017; Shi et al. 2022). Crucially, some of the transgenic lines showed a complete absence of TALENs sequences in downstream characterization, offering a transgene-free approach (Clasen et al. 2016).

CRISPR/Cas technology is applied to reduce the reducing sugar content and mitigate acrylamide formation during frying by targeting *VInv *expression (Yasmeen et al. 2022; Shi et al. 2022; Jaiswal 2023). The sucrose involved in CIS is transported into the vacuole through the tonoplast sugar transporter (TST) (Martinoia 2018). Silencing *StTST1 *increases sucrose in cytoplasm, furthermore reduces starch degradation and reducing sugar accumulation in tubers, effectively enhancing resistance to CIS (Liu 2023). Recently, two sgRNAs were applied in the CRISPR-Cas9-mediated knockdown of vacuolar invertase (*VInv*) gene in local cultivar of potato in Pakistan. And the overall editing efficacy was determined to be 25.6% and the content of reducing sugars showed five-fold reduction than control (Yasmeen et al. 2022). This is the first successful application of potato *VInv* gene's knockdown in addressing cold-induced sweetening by resulting in minimum accumulation of reducing sugars in genome-edited potato.

### Reducing enzymatic browning

High tuber quality is a crucial trait valued by both the potato-processing industry and consumers. However, throughout harvesting, transportation, and processing, enzymatic browning (EB) can occur on damaged tissue surfaces, severely affecting the flavor, appearance, and nutritional value of potato products. Moreover, mechanical damage disrupts subcellular compartments, releasing phenolic compounds localized in vacuoles and polyphenol oxidases (PPOs) localized in amyloplasts (Thygesen 1995). PPOs catalyze the oxidation of monophenols and/or o-diphenols to o-quinones in the presence of oxygen, which then react with free amino acids and macromolecules such as proteins, forming red or brown pigments that accumulate in plant tissues (Boeckx et al. 2015; Taranto et al. 2017). Various chemical methods exist to prevent enzymatic browning in potatoes, primarily based on inhibiting PPO activity (Moon et al. 2020). However, due to growing concerns about chemical residues impacting public health, there is increasing interest in cultivating potato varieties resistant to enzymatic browning.

PPOs comprise a multi-gene family with diverse expression patterns. In potatoes, five *PPO *genes have been identified, each with several allelic variations (Thygesen 1995). Knocking out all *PPO *genes could potentially be lethal for plants, given their involvement in numerous physiological processes and defense mechanisms against pathogens and pests. Hence, precise allele silencing or gene targeting is necessary. PPO2 is the primary isoform responsible for 55% of total PPO activity in tubers (Chi 2014). Successful mutation of the *StPPO2 *gene was achieved in the cv. Désirée through CRISPR/Cas9 RNP delivery to protoplasts (González et al. 2020). Among the regenerated plants, 68% displayed mutations in at least one allele of the target gene, with 24% exhibiting mutations in all four alleles. As anticipated, these lines showed reduced PPO activity and enzymatic browning compared with control plants, with no observed off-target mutations in the remaining StPPO genes, suggesting that enzymatic browning can be significantly reduced by editing a single StPPO member. Thus far, obtained individuals with low PPO activity through gene editing without showing a significant impact on their growth and development, indicating the feasibility of obtaining anti-enzymatic browning potato varieties through *PPO* genes modification.

### Reducing the content of glycoside alkaloids in tubers

Glycoalkaloids are secondary metabolites found in *solanaceous *plants. Steroidal glycoalkaloids (SGAs), primarily composed of α-solanine and α-chaconine in commercial cultivars, are typically concentrated in flowers, sprouts, leaves, and especially in the peel of tubers (Friedman 2006). The SGA content in tuber flesh can increase substantially under wounding, mechanical stress, or exposure to high light during post-harvest handling and storage (Ginzberg et al. 2009). Accumulation of SGAs is toxic to humans and imparts a bitter taste. RNAi-mediated silencing of the host gene *glycoalkaloid metabolism 4* (*GAME4*), involved in the SGA biosynthetic pathway, resulted in a significant decrease (up to 74-fold) in SGA content in leaves and tubers (Itkin et al. 2013). Similarly, lower SGA content was achieved through the RNAi silencing of glycoalkaloid metabolism 1 (GAME1) gene (Cárdenas et al. 2016).

*St16DOX*, encoding a steroid 16α-hydroxylase crucial for SGA biosynthesis and existing in a single copy, suppressed SGA accumulation in potato hairy roots through CRISPR/Cas9 targeting (Nakayasu et al. 2018). Among the 25 transgenic hairy root lines, two contained no detectable α-solanine and α-chaconine, instead accumulating 22,26-dihydroxycholesterol, an St16DOX substrate, suggesting the complete disruption of *St16DOX*. However, whole plants have not yet been regenerated.

*Sterol side chain reductase 2* (*SSR2*), responsible for cholesterol synthesis, was targeted using TALEN (Sawai et al. 2014). Only one stable line with tetra allelic mutations in gene *SSR2*, lacking a wild-type copy, exhibited significantly reduced SGA content (10% that of the wild type) in leaves. However, SGA content in tubers of this line was not reported. In a recent study, SSR2 was targeted using CRISPR/Cas9, but tetra allelic mutants could not be obtained (Zheng et al. 2021). Although reductions in SGA content up to 34% in tuber flesh were reported, some lines exhibited higher SGA levels in both tubers and leaves compared with the wild type.

### Overcoming self-incompatibility in potato

Cultivated potatoes are primarily tetraploid, which poses challenges for improvement due to tetrasomic genetics and clonal propagation. There is increasing interest in re-domesticating potatoes as an inbred line-based crop propagated by seeds at the diploid level (Jansky et al. 2016). However, the self-incompatibility (SI) and inbreeding depression of diploid potatoes substantially hinder the development of inbred lines. In potato, self-incompatibility is governed by the single, multi-allelic S-locus comprising the S-locus RNase (S-RNase) and S-locus F-box proteins (SLFs). The S-RNase protein recognizes self-pollen and inhibits its elongation through RNA degradation (Kubo et al. 2010). Thus, inhibiting the function of S-RNase could potentially yield self-compatible lines. Two studies have independently targeted conserved regions of S-RNase alleles to knock out this gene using CRISPR/Cas9 (Enciso-Rodriguez et al. 2019; Ye et al. 2018). The resulting mutants harbored multi-allelic mutations in the S-RNase gene and transmitted self-compatibility to their progeny. Moreover, in some cases, the *Cas9* gene segregated out in T1 seeds, suggesting the possibility of obtaining nontransgenic progeny. Furthermore, the function of the *S-locus inhibitor* (*Sli*) was explored by targeting it with Cas9 to convert self-compatible varieties into self-incompatible ones (Eggers et al. 2021). Double knockout lines of HT-B and S-RNase displayed increased seed production up to three times higher than observed in the S-RNase-only knockout, indicating a synergistic effect between HT-B and S-RNase in self-compatibility in diploid potato (Lee et al. 2023). These studies introduced a new approach to diploid potato breeding, expanded the resources of self-compatible potatoes, and are poised to accelerate genetic improvements in potatoes.

### Enhancing biotic stress resistance in potato

Traditionally, fungicides have been used to protect against late blight, but this approach can lead to chemical pollution. Compared with chemical pesticides, planting disease-resistant varieties remains the most cost-effective and environmentally friendly method for preventing and controlling potato late blight. Resistance to *P. infestans* is conferred by dominant *R* genes encoding proteins that recognize avirulence (Avr) effectors and trigger plant responses. Incorporating *R*genes from wild potato relatives is currently considered the most reliable and environmentally friendly approach (Stefańczyk et al. 2020). Successful transfer of *R *genes has been achieved (Halterman et al. 2016; Ghislain et al. 2019; Byarugaba et al. 2021), resulting in genetically modified cultivars resistant to widespread *P. infestans *infection. These cultivars are primarily used for cultivation in sub-Saharan Africa (Ghislain et al. 2019; Byarugaba et al. 2021).

CRISPR/Cas-based technologies have ushered in a new era for exploiting plant resistance to late blight and deciphering the mechanisms behind plant development and immunity even in food crops by two main strategies: knockout of susceptibility genes and knock-in of metabolite biosynthetic genes (Hegde et al. 2021; Kieu et al. 2021; Ma et al. 2023). In the knockout approach, (Kieu et al. 2021) targeted potato susceptibility genes (*S*-genes) triggered upon infection by *P. infestans*. Seven putative potato *S*-genes were analyzed in cvs. Désirée and King Edward. Tetra-allelic deletion mutants were assessed for resistance to *P. infestans* based on lesion size, percentage of infected leaves, and seedling morphology. Three *S*-genes, *StDND1*, *StCHL1*, and *StDMR6-1*, were implicated in *P. infestans* susceptibility. Mutant plants lacking *StCHL1* and *StDMR6-1* exhibited late blight resistance, with reduced lesion size compared with WT and unaffected morphology. These lines with loss-of-function mutations in S-genes could be valuable in breeding to confer resistance to *P. infestans*, particularly when combined with specific R-genes.

In the knock-in approach, resistance to *P. infestans* in cv. Russet Burbank was enhanced by identifying a pathogen-responsive gene, *StCCoAOMT*, through RNA-seq analysis in both mock and *P. infestans*-inoculated potato plants (Hegde et al. 2021). *StCCoAOMT *encodes caffeoyl-CoA methyltransferase, an enzyme involved in synthesizing defense-related metabolites in plants (Hegde et al. 2020). A specific allele of *StCCoAOMT* harboring a point mutation was identified, which generates a premature termination codon and results in a truncated protein lacking 96 amino acids from the N-terminus. Recently, Cas9-mediated HDR was employed to replace an SNP in the *StCCoAOMT *gene, eliminating the premature stop codon and restoring the full-length protein (Hegde et al. 2021), leading to increased expression of downstream*R*-genes and imparting partial late blight resistance in transgenic plants. Notably, this resistance was characterized by a significant reduction in plant severity and pathogen biomass (more than a 21-fold decrease) in stems. An increased accumulation of feruloylated metabolites, involved in tuberization and lignification of cell walls, was also observed around the infection site.

Another destructive pathogen affecting potatoes is PVY, causing substantial losses in both tuber quality and quantity, up to 80% yield loss (Quenouille et al. 2013). Because PVY is transmitted by various aphids, reducing vector populations through insecticide use is a common method to prevent virus transmission. However, the most effective approach is developing resistant cultivars through genetic engineering. Recently, resistance to multiple PVY strains was achieved through the knockout of viral RNA transcripts by using the CRISPR/Cas13a system. sgRNAs were designed from conserved coding regions of three PVY strains (PVY^O^, PVY^N^, and the recombinant PVY^N:O^ strain), targeting proteins P3 (viral factors), CI (virus movement), Nlb (viral replicase), and CP (capsid) regions of the viral genome. All potato lines with Cas13a/sgRNA targeting P3, CI, Nlb, and CP in the PVY genome exhibited the ability to cleave the RNA genome of PVY. Moreover, they displayed no disease symptoms and a considerable reduction in PVY^O^ and PVY^N^accumulation in leaves, with symptom severity negatively correlated with LshCas13a/sgRNA expression levels (Zhan et al. 2019). This broad-spectrum resistance to different potato viruses (such as PVS, PVY, and PVA) can be engineered using the same multiplexing method. A recent study targeted eIF4E1, a host protein exploited by PVY for replication after infecting cells, thus providing recessive resistance when mutated (Lucioli 2022). The first generation of transgenic potatoes had only two eIF4E1 allele mutations, rendering them susceptible to PVY^NTN^. Regeneration and re-transfection of protoplasts isolated from the first generation of mutant plants produced two complete KO lines with partial resistance to PVY^NTN^ infection.

### Advances in genome studies of potato

#### Identification, collection and evaluation of germplasm resources

Germplasm resources are the foundation of modern seed industry. The collection, identification, and evaluation of excellent germplasm resources are prerequisites for variety breeding and elite gene mining. According to data provided by the World information and early warning system on plant genetic resources for food and agriculture (WIEWS, https://www.fao.org/wiews/zh/), there are currently 82,293 potato germplasm resources preserved in 89 institutions and 4 international/regional research centers in 59 countries worldwide. These collections are of great significance for the study of potato germplasm diversity and crop improvement, and provide valuable resources for researchers.

In potato breeding, tuber morphological traits are important selection targets for the demands of the fresh and processing markets. Potato varieties with increased levels of minerals have great potential to alleviate mineral malnutrition. A diversity panel of 214 advanced clones was genotyped and phenotyped to obtain genomic estimated breeding values, which helps to better understand the genetic basis of potato morphological traits and mineral macro and micronutrients, and identify parents with the best breeding values to improve selection efficiency (Pandey et al. 2023a, b). (Singh et al. 2022) evaluated the mineral nutrient content of 243 tetraploid potato tubers with and without skin, and found that the nutrient concentration in the epidermal layer of the tubers was high and peeling would lead to nutrient loss, providing a reference for breeding high nutrient potato varieties.

To gain insight into the genetic potential of the germplasm used for potato breeding in a Nordic breeding program as well as all available accessions from the Nordic genebank (NordGen), a genotyping and trait evaluation on 133 breeding backbone parents and gene banks was conducted, suggesting that more genotypes outside of the Nordic region should be introduced in the subsequent breeding process (Selga et al. 2022). Another phenotypic variation and genetic diversity of 149 main potato cultivars in China identified a molecular marker STI032 that is significantly correlated to starch content and maturity (Hu et al. 2022).

The resistance of 189 late blight resistant varieties was evaluated, and identified 10 elite broad-spectrum resistance resources and 127 Phytophthora infestans resistance resources, revealing the rich genetic diversity of wild resources (Duan et al. 2021). With the development of molecular biology technology, the gradual deepening of understanding of potato germplasm resources at the molecular and genomic levels will provide abundant information for genetic study and breeding, thereby effectively utilizing germplasm resources and accelerating the potato genetic improvement and new germplasm innovation.

#### Genome/high-throughput sequencing provides genetic resources/gene targets

A profound understanding of potato genomics and genetics is essential for effective molecular breeding. High-quality genome sequence data are fundamental for identifying key genetic factors in potato breeding, such as self-incompatibility, inbreeding depression, and the genetic basis of tuber formation. Since the release of the first reference genome for a monoploid potato (DM1-3 516 R44) in 2011, several studies have reported the genome assemblies for various cultivated and wild diploid potato accessions, offering valuable resources (Potato Genome Sequencing Consortium 2011; Aversano et al. 2015; Leisner et al. 2018; Zhou et al. 2020; Yan et al. 2021; Tang et al. 2022). However, most potato cultivars have tetraploid genomes, presenting a significant challenge for genome assembly. Existing tetraploid genome sequences are limited to whole-genome shotgun sequencing data (Hardigan et al. 2017) or highly fragmented assemblies (Kyriakidou et al. 2020), lacking phased haplotype data for the four alleles.

Recent advances in third-generation sequencing technology, coupled with improved data computing and analysis capabilities, have enabled comprehensive analysis of autotetraploid potato genomes using long-range, high-accuracy DNA sequencing (Cheng et al. 2021). A combination of PacBio 3.1 HiFi and single-cell sequencing technology, along with HiC technology-assisted assembly was applied and obtained four sets of haplotype genomes for the tetraploid variety Otava, yielding important reference data for tetraploid potato research including the annotation of 152,855 genes, with 54% present in all four haplotype genomes (Sun et al. 2022b). Wang et al. (2022a, b) presented a high-quality, chromosome-scale reference genome sequence of Qingshu 9, a homotetraploid heterozygous potato variety. Leveraging cutting-edge sequencing technologies and polyploid graph binning, Bao et al. (2022) achieved a chromosome-scale, haplotype-resolved genome assembly of the cultivated potato Cooperation-88 (C88), facilitating research on potato genome variation.

The concept of the pan-genome, encompassing all genetic information of a species, offers a broader view of genetic diversity than a single reference genome. Hoopes (2022) generated phased genome assemblies of six potato cultivars, cataloging the gene complement, identifying allelic variation associated with agronomic traits, and constructing the first pan-genome for tetraploid potato. Furthermore, Tang et al. (2022) conducted evolutionary analysis of 432 diploid materials, constructing high-quality diploid potato genomes and pan-genome maps for cultivated and closely related wild species. These genome disclosures, including those of tetraploid cultivars such as Atlantic, Otava, Qingshu 9, and C88, provide unprecedented genetic insights into genomic structure, variation, and diversity, laying a solid foundation for variety improvement based on genomic variation.

#### GWAS for potato

Genome-wide association study (GWAS) has proven effective in identifying causal variation of complex traits, offering higher resolution at the genome level compared with traditional linkage mapping strategies (Naeem et al. 2021). Sharma (2018) examined various GWAS models in cultivated potato genotypes using the Infinium 8 K Potato SNP Array and found that kinship, not population structure, was the most important factor in determining the extent of false associations. Using the tetraploid potato genome, GWAS has been employed to associate candidate genes with specific chromosomes in cultivated potato species for protein content (Klaassen et al. 2019), mineral content (Pandey et al. 2023a), scab resistance (Kaiser et al. 2020), root and stolon traits (Yousaf 2021), tuber traits (Pandey et al. 2022), tuber bruising (Angelin-Bonnet et al. 2023), and tuber-bound free amino acids (Pandey et al. 2023b). Re-sequence analysis on 214 representative potato varieties bred in the United States was conducted and uncovered regulatory genes linked to crucial traits such as skin color, growth period, and tuber formation (Pandey et al. 2021). Similarly, nine related regions and three candidate genes associated with drought stress were identified through GWAS analysis (Díaz et al. 2021).

In the field of disease resistance, Wang et al. (2021) analyzed 284 tetraploid potatoes via GWAS, identifying 44 candidate genes linked to late blight. Zhang et al. (2022) re-sequenced the genomes of 108 core cultivar potato accessions with rich genetic diversity and population structure from the International Potato Center, revealing numerous candidate loci related to photoperiodic flowering time and temperature sensitivity through GWAS, which provides a valuable resource facilitating the understanding of the domestication process, genetic studies, and agronomic improvement of autotetraploid potato. A GWAS on the tuber flesh color of 150 tetraploid heterozygous potatoes was conducted and identified the associated candidate genes (Wang et al. 2022a, b). These GWAS findings indicate the significance of high-quality reference genomes in analyzing complex genomes and establish a foundation for further research on genetic analysis and variety selection for crucial traits.

#### Genomic design and breeding technology for diploid hybrid potato

Tubers are the planting material in commercial potato cultivation. However, clonal propagation leads to low reproduction coefficient, high cost of storage and transportation, whereas tubers are easy to carry viruses and pests, which have hindered the development of potato industry for a long time. Unlike the modern cultivation of potatoes, which mainly rely on tetraploids, approximately 70% of potato germplasm resources in nature are diploid (Spooner et al. 2014). Therefore, screening and identifying excellent diploid potato germplasm and developing a hybrid potato breeding system aimed at converting potato from a tuber-propagated tetraploid crop into a seed-propagated diploid crop through crossing inbred lines, has become a research hotspot in the potato breeding and research community (Lindhout et al. 2011; Jansky et al. 2016). However, achieving diploid hybrid potato breeding is not an easy task, as it requires highly homozygous inbred parents to cross and exhibit heterosis, while the inherent self-incompatibility and inbreeding depression of natural diploid potato germplasm hindered the development of high-purity and excellent inbred lines.

To cultivate inbred lines, the first step is to solve the problem of incompatible inbreeding. Qiao et al. (2004a, 2004b) used plants such as *Antirhonum majus* and *Petunia* hybrid as model to elucidate the molecular mechanism by which the S-RNase gene determines self-incompatibility, laying a theoretical foundation for breaking the limitation of self-incompatibility in diploid potatoes. Additionally, comparative genomics methods combined with genome editing techniques were applied to two naturally mutated self-compatible clones from wild potatoes and the genes controlling this trait were identified, *S-RNase* and *Sli* (*S-local inhibitor*) (Zhang et al. 2019). Subsequently, by cloning *Sli *gene in the pollen, cross of self-compatible diploid potato RH89-039–16 with self-incompatible lines was revealed to induce the mating transition from self-incompatibility to self-compatibility effectively (Ma et al. 2021). Breaks self-incompatibility in diploid potatoes paves a path forward for the diploid hybrid breeding program (Eggers et al. 2021).

Inbreeding depression refers to the phenomenon of decreased vitality, weakened resistance, and reduced yield in the offspring of biological self-pollination. Potatoes accumulate a large number of recessive deleterious mutations during long-term asexual reproduction. Once self-pollinated, the effects of deleterious mutations will become apparent in offspring, leading to inbreeding depression. Recently, the extensive deleterious mutations in a diploid potato diversity panel were identified, providing new evidence for identifying functional sites and eliminating harmful mutations, and facilitating developing strategies to minimize fixed load in breeding populations (Wu et al. 2023). Moreover, the effect of deleterious mutations in hybrid varieties was revealed to be masked by crossing inbred lines with significant genetic background differences (Zhang et al. 2019). However, the deleterious mutations that lead to inbreeding depression are embedded in the two haplotypes of potatoes and cannot be completely eliminated through recombination (Zhou et al. 2020). Therefore, it is necessary to select, design, and eliminate deleterious mutations, overcome inbreeding depression, and obtain excellent purebred inbred lines with the genomic information.

Recently, a hybrid potato genome design system has been established and highly viable and fertile inbred lines (with a homozygosity of up to 99.94%) were obtained from different inbred lines, and hybridized two different inbred lines to produce the first generation of identical F1 hybrid H1. A plot trial showed that the estimated tuber yield of the F1 hybrid H1 was close to 40 tons per hectare, displaying strong heterosis (Zhang et al. 2021). Though potato hybrid breeding is still in its infancy, it has been a milestone achievement, opening a door for potato breeding and ushering in the era of precision breeding and rapid iteration for genetic improvement of potatoes (Markel and Shih 2021; Mascher et al. 2021).

#### Future perspectives

With traditional breeding techniques forming the backbone of potato variety selection globally, the breeding process tends to be lengthy. However, with rapid advancements in biotechnology and sequencing technology, significant strides have been made in potato basic research. These advancements can advance the understanding of genetic mechanisms underlying crucial traits and hold promise for enhancing comprehensive breeding technologies. The successful assembly of haplotype genomes for tetraploid cultivated species and the construction of variation and pan-genome maps for diploid wild and cultivated species mark pivotal breakthroughs in molecular breeding and whole-genome selection breeding. Leveraging the research advantages of pan-genomics and identifying key traits such as agronomy, quality, stress resistance, and disease resistance regulatory genes, as well as analyzing genetic regulatory mechanisms and molecular networks, will significantly boost the evaluation and utilization of germplasm resources and the genetic enhancement of potato varieties.

The use of CRISPR/Cas gene editing technology for variety enhancement and the development of superior new varieties presents a rapid and effective approach, albeit with challenges such as the simultaneous mutation of four alleles. Gene knockout is the primary method for potato gene editing, with the less commonly used, more precise editing tools such as Single Base Editor and Prime Editor facing hurdles, primarily due to their relatively low editing efficiency in potatoes. However, these novel tools require specific protocols and enhancements tailored to the potato context. Developing a genotype-independent genetic transformation system, enhancing genetic transformation efficiency, and broadening the application scope of CRISPR/Cas technology are crucial pathways for improving potato variety genetics. Additionally, prioritizing tools that do not leave traces of exogenous DNA is essential, given their lower regulatory barriers. With the ongoing sequencing of more tetraploid potato genomes and the refinement of gene editing technology, a wider array of genes will become available for precise and efficient breeding of new potato varieties in the future.

## Data Availability

All relevant data are within the manuscript.

## Abbreviations

GA: Gibberellic acid
CKs: Cytokinins
ET: Ethylene
ABA: Abscisic acid
IAA: Indole-3-acetic acid
BRs: Brassinosteroids
SA: Salicylic acid
SLs: Strigolactones
*ARF*: Auxin response factor
*CKX1*: CK oxidase/dehydrogenase
RNA-seq: RNA-sequencing
2-DE: Two-dimensional electrophoresis
LC-Triple TOF MS/MS: Reversed-phase liquid chromatography-tandem mass spectrometry
ClO_2_: Chlorine dioxide
AI: Alkaline invertases
NI: Neutral invertases
*GA2ox*: GA2-oxidase
RSA: Root system architecture
ARs: Adventitious roots
LRs: Lateral roots
N: Nitrogen
Mg: Magnesium
P: Phosphorus
*ABI5*: ABA insensitive 5
*bZIP*: Basic leucine zipper
*StABL1*: StABI5 like 1
*StAST1*: *StABL1* And *StSP6A*-associated TCP protein 1
aTAC: Nodulin-activated complex
SAM: Shoot apical meristem
IM: Inflorescence meristem
*FT*: FLOWERING LOCUS T
CO: CONSTANS
RFT1: RICE FLOWERING LOCUS T1
FAC: Flowering activation complex
aTACs: Alternative TACs
aFACs: Alternative FACs
BBX: B-box type zinc finger
PHYA: Phytochrome A
PHYB: Phytochrome B
PHYF: Phytochrome F
DLI: Daily light integral
*SUT*: Sucrose transporter
RNAi: RNA interference
SGAs: Steroidal glycoalkaloids
SES: SUPPRESSING EXPRESSION OF SP6A
*CKX2*: CYTOKININ OXYDASE 2
CCM: Chloroethyltrimethylammonium chloride
FKF1: FLAVIN-BINDING KELCH REPEAT F-BOX PROTEIN 1
GI: GIGANTEA
DOF: DNA-binding with one finger
m6A: N6-Methyladenosine
F6P: Fructose 6-phosphate
PGI: Phosphoglucoisomerase
PGM: Phosphoglucomutase
AGPase: ADP-glucose pyrophosphorylase
SuSy: Sucrose synthase
SSs: Starch synthases
GBSSs: Granular-bound starch synthases
SBEs: Starch branching enzymes
DBEs: Debranching enzymes
GBSS: Granule-bound starch synthase
PTST: PROTEIN TARGETING TO STARCH
PWD: Phosphoglucan, water dikinase
GWD: Glucan, water dikinase
BAM: Beta-amylase
DPE: Disproportionating enzyme
SBE: Branching enzyme
FW: Fresh weight
ELIs: Elicitins
RLP: Receptor-like protein
ELR: ELICITIN RESPONSE
SOBIR1: SUPPRESSOR OF BIR1-1
RLKs: Receptor-like kinases
*SRF*: STRUBBELIG-RECEPTOR FAMILY
LecRK-I.9: L-type lectin receptor kinase-I.9
PERU: Pep-13 receptor unit
CRN: Crinkler
PR1: Pathogenesis-related protein-1
BSL1: BSU-LIKE PROTEIN1
StDeSI2: Desumoylating isopeptidase 2
NRL1: NPH3/RPT2-LIKE1 protein
PP1c: Protein phosphatase 1 catalytic
PVY: *Potato virus Y*
PVX: *Potato virus X*
PVS: *Potato virus S*
PVA: *Potato virus A*
PLRV: *Potato leafroll virus*
PVM: *Potato virus M*
HR: Hypersensitive resistance response
ER: Extreme resistance
CP: Coat protein
PD: Plasmodesmata
PM: Plasma membrane
REM: REMORIN
TGBp1: Triple gene block proteins1
*ICE1*: INDUCER OF CBF EXPRESSION 1
StTOC1: TIMING OF CAB EXPRESSION1
*DOG1*: DELAY OF GENEATION 1
*SLP*: SUBTILISIN-LIKE PROTEASE
*AtHKT1*: *Arabidopsis* High-affinity potassium transporter 1
SnRK2: Sucrose nonfermenting 1-related protein kinases 2
NF-Y: Nuclear factor Y
RNAi: RNA interference
PLRV: Potato leafroll virus
SSNs: Site-specific nucleases
ZFNs: Zinc Finger Nucleases
TALENs: Transcription Activator-Like Effector Nucleases
CRISPR: Clustered Regularly Interspaced Short Palindromic Repeats
CRISPR/Cas: CRISPR-associated proteins
RNP: Ribonucleoprotein
CBE: Cytidine base editor
SBE: Starch branching enzymes
CIS: Cold-induced sweetening
VInv: Vacuolar invertase
asRNA: Antisense RNA
TST: Tonoplast sugar transporter
EB: Enzymatic browning
PPOs: Polyphenol oxidases
SGAs: Steroidal glycoalkaloids
*GAME4*: Glycoalkaloid metabolism 4
*SSR2*: Sterol side chain reductase 2
SI: Self-incompatibility
S-RNase: S-locus RNase
SLFs: S-locus F-box proteins
*Sli*: S-locus inhibitor
